# Advancements in Methods and Camera-Based Sensors for the Quantification of Respiration

**DOI:** 10.3390/s20247252

**Published:** 2020-12-17

**Authors:** Haythem Rehouma, Rita Noumeir, Sandrine Essouri, Philippe Jouvet

**Affiliations:** 1 École de Technologie Supérieure, Montreal, QC H3T 1C5, Canada; rita.noumeir@etsmtl.ca; 2 CHU Sainte-Justine, Montreal, QC H3T 1C5, Canada; sandrine.essouri.hsj@ssss.gouv.qc.ca (S.E.); philippe.jouvet@umontreal.ca (P.J.)

**Keywords:** breathing monitoring, camera-based monitoring systems, respiratory physiological variables, chest wall deformities, depth sensors

## Abstract

Assessment of respiratory function allows early detection of potential disorders in the respiratory system and provides useful information for medical management. There is a wide range of applications for breathing assessment, from measurement systems in a clinical environment to applications involving athletes. Many studies on pulmonary function testing systems and breath monitoring have been conducted over the past few decades, and their results have the potential to broadly impact clinical practice. However, most of these works require physical contact with the patient to produce accurate and reliable measures of the respiratory function. There is still a significant shortcoming of non-contact measuring systems in their ability to fit into the clinical environment. The purpose of this paper is to provide a review of the current advances and systems in respiratory function assessment, particularly camera-based systems. A classification of the applicable research works is presented according to their techniques and recorded/quantified respiration parameters. In addition, the current solutions are discussed with regards to their direct applicability in different settings, such as clinical or home settings, highlighting their specific strengths and limitations in the different environments.

## 1. Introduction

**Figure 1:**
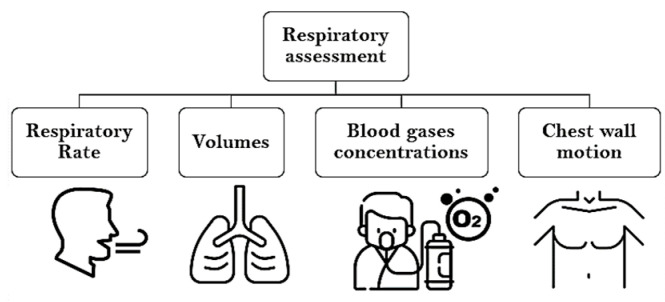
Respiratory parameters classification.

Breathing monitoring is an important component of clinical detection of vital distress, and is performed for all patients in a hospital, whether in the emergency room, a general or specific ward, or in the intensive care unit. The monitoring of a patient respiration mainly comprises an assessment of the chest wall motion [1,2,3,4] and measurement of physiological parameters, such as airflow and respiratory volumes [5,6,7,8,9], respiratory rate (RR) [10,11,12,13], the resulting transcutaneous oxygen saturation (SpO_2_), and respiratory CO_2_ removal [14,15,16,17,18]. Respiration assessment can be classified around these four elements, as shown in Figure 1. 

Chest wall motion assessment and physiological parameter monitoring are two essential caregivers’ clinical daily tasks. They are key elements in managing patients with respiratory failure, whether this failure is due to chronic illness or to an acute pathology in the lungs, airway, or muscles, and to assess the impact of a therapy. Therefore, increased efforts have been made in developing effective respiratory measurement systems with action- and results-oriented goals, paving the way to new lines of research. These new research avenues have led to cooperative efforts between physicians and engineers, helping to propose new methods and systems for respiration quantitative assessment. 

Existing measurement systems, while diverse, fall into two broad groups: contact and non-contact [19]. Traditional techniques of measurement are based contact, use a large number of sensors, require direct contact with the equipment, rely on patient cooperation, and require staff that is sufficiently skilled and trained to manage the measurement systems and use them correctly [20]. The most widespread techniques are plethysmography [21] and spirometry [22]. These approaches have been promoted and deployed in hospital wards. Nevertheless, they may easily disturb the patient from a clinical perspective, especially in younger children, frail elderly, and cognitively impaired patients. 

Plethysmography, for instance, requires connection to the patient using many wires, some attached to the facial area [19]. This is often uncomfortable and may be poorly tolerated in children. In addition, patient movements may lead to a high rate of errors in the measurements [23]. Likewise, the spirometer, although considered as the gold standard for pulmonary function testing, the measurement validity depends upon many factors, such as gas temperature, humidity, viscosity, and density. It needs to be recalibrated at least once every two days [24]. In addition, spirometry requires a high level of cooperation not achievable in infants and toddlers, elderly people, and in acute care [25,26]. Indeed, the subject is requested to have a big breath and then exhale the air through a mouthpiece attached to the spirometer, while having the nose pinched off. This highlights another important problem with spirometry, which is that critically ill patients could not endure a mouthpiece or a facemask [27]. Moreover, the whole process may interfere with the true natural respiratory activity of the patient (due to stress, unease, etc.). The reported consequences on respiration are falls in the breathing rate, increases in the tidal volume and amplified CO_2_ production. Perez et al. have reported that forced oral respiration may be causing collateral effects on respiratory activity [28].

Consequently, non-contact methods have been of big interest to researchers and clinicians. These methods aim to reduce or even eliminate the large number of sensors attached to a patient’s body to monitor breathing. 

The advantages of non-contact methods include reducing system complexity, improving their portability, increasing the flexibility so they can be used by almost anyone (including non-trained staff) and facilitating the data collection process [19,29]. The trend today shows that non-contact systems as very powerful, but still less mature then contact ones, primarily in clinical environments. Concerns related to patient safety, electromagnetic interference with other electronic devices, rapid interconnection, and integration with existing medical equipment and the complexity of the early versions, which usually were not optimized to be used daily, are all parameters in their slothful uptake in hospitals. With further progress, non-contact methods will become more and more viable, and will have a strong growth potential. However, at the present stage of their evolution, there is an obvious need to improve the effectiveness of these systems to meet their expected performance in the different environments [30,31,32]. This requires a definition of the challenges and opportunities facing the proposed solutions and motivating this line of research.

In this review, we present the current approaches for breathing assessment across many fields. The potential advantages of using non-contact systems are emphasized. The strengths and limitations of the different approaches are discussed, highlighting their performances in various settings and considering the users’ expectations, including medical staff, patients, athletes, and normal users for home applications.

### 1.1. Related Work

Recently, several studies have reported developments in respiration assessment, which includes both chest wall motion and physiological parameter estimation. The problem of respiratory motion has been studied through a comprehensive review, published by McClelland et al. [33]. In this work, authors proposed to build breathing models to compensate for the motion of internal organs in radiotherapy and image-guided interventions. Using a respiratory motion model, estimates of breathing signals can be derived from other input signals, or from surrogate data. Examples of surrogate data include the displacement of the skin surface, the spirometer signal, the respiratory belt, and data acquired through imaging techniques, such as magnetic resonance imaging (MRI), ultrasound (US), and computed tomography (CT). The breathing motion entails non-negligible variations between and within respiratory cycles, such as amplitude changes. These changes can be broadly categorized into two main classes: intra-cycle variation and inter-cycle variation [33]. The first category considers the variations of the motion path between the inspiration and expiration of each cycle, while in the second category, only variations between breathing cycles are considered. To model these variations, the researchers used the respiratory phase, in which they assumed that breathing was periodic. However, this may be not effective in critically ill patients who often present irregular breathing. Additionally, the focus was on modeling the respiratory motion, especially for radiotherapy and image-guided intervention applications. Authors described the work conducted to describe and track the movement of body parts/organs due to respiration. Moreover, the review did not present methods aiming to quantify physiological variables such as respiratory rate and tidal volume.

There have been several studies reporting techniques for estimating physiological parameters.

For instance, Al-Khalidi et al. reviewed methods measuring respiratory rate [19]. They classified the existing techniques into two main categories: contact- and non-contact-based. The described contact techniques include nasal prongs, masks, thermistors, microphones, electrocardiogram (ECG) electrodes, respiratory impedance plethysmography, pulse oximeter, and electrodes diffusing gas in a patient’s skin. The described non-contact techniques were mainly based on radar, infrared and optical imaging techniques. Other non-contact techniques were briefly reported, such as three-dimensional (3D) vision algorithms and webcam cameras. Moreover, the authors only describe techniques for the respiratory rate parameter and not for other physiological parameters, such as volumes.

Kim et al. [34] provided a list of methods for human breath analysis, in which exhaled breath is tested for the evaluation of health status and disease types. However, their review fits more into clinical diagnosis. Methods for breathing monitoring in clinical environments were also reviewed by Folke et al. [35]. Their work classified non-invasive methods and devices that provide measures for respiratory rate, tidal volume, and gas exchange. The methods’ merits and limitations are also discussed. Although the review offers a good categorization of devices and monitoring principles, many new methods and devices have been developed since the review was produced. Advances in imaging technology over the past ten years, and especially with the emergence of new depth acquisition devices with acceptable accuracy [36,37,38,39,40,41], have paved the way to new non-contact methods, which were not addressed in their review.

Recently, Massaroni et al. [42] presented a review for contact-based respiratory rate techniques. Authors presented the sensors’ working principles, metrological characteristics, and their major applications in the respiratory monitoring field. However, the focus was on what contact-based sensors can offer for respiratory rate measurement. Hence, there were no recommendations or discussions related to contactless-based methods and their suitability in measuring respiration in different settings. Moreover, the review did not cover methods aiming to quantify volumes or other respiratory variables.

### 1.2. Paper Contributions and Structure

This paper presents a review of the latest respiratory assessment methods and systems, with a focus on non-contact devices. Camera-based systems are highlighted. Their direct applications are discussed in a variety of settings (i.e., clinical settings, home, prisons, cars, exercise, etc.). Through this work, we emphasize other challenges with actual research works. The main contributions of our work are:

- An update of the literature covering assessment of human breathing [19,33,34,35,42] with the latest works, more recently published meta-analyses and new challenges and perspectives.
- Categorization based on the sensor’s technology.
- Identification of the current needs and prospects in various lines of research and remarks for future work.
- Surveying advances made in the latest non-contact devices and camera-based monitoring systems.

## 2. Search Methodology

Following the requirements of Multidisciplinary Digital Publishing Institute (MDPI) *Sensors*, a systematic review following the Preferred Reporting Items for Systematic Reviews and Meta-Analyses (PRISMA) guidelines [43] was conducted. A total of five databases were searched, including: Scopus, ScienceDirect, IEEE Xplore, PubMed, and Web of Science. The following search phrases were used: ((Respiratory rate OR Volume OR Respiratory Motion) AND (Assessment OR Measurement OR Evaluation) AND (Respiration OR Breathing) AND (Systems OR Cameras OR Sensors)). Other records were identified through resources like Google Scholar search, which allow finding some works not found in the other databases. A combination of the following strings has been used as well: “Respiratory assessment”, “Respiratory monitoring”, “Respiratory rate”, “Volumes”, “Chest wall assessment”, “Respiration Motion”, “Respiration pattern”, “Breathing rate”, “Vital signs”, “Intensive care environment”, “Non-contact methods”, “Breathing disorders”, “Kinect Camera”, “Depth sensors”, “Gas exchange”, “Carbon dioxide partial pressure”, “Oxygen partial pressure”, “Tidal volume”, “Respiratory distress”, “Newborns”, “Infants”, “Adults”, “Surface reconstruction”, RGB cameras”, “RGB-D cameras”, “Thermal cameras”, “Radar”, “Time-of-Flight”, “Structured light”, “Active stereoscopy vision”, “3D technologies”, “Consumer grade camera”, “Measurement”, “Measurement”, “Measurement”. During the search, the keywords were combined with each other to broaden or narrow the search results. Moreover, the terms in the search phrase were replaced with the cited strings to create variations of this query.

The searches were conducted between 1 March 2020 and 31 October 2020. The publication period investigated was from 2010 to 2020. We used the setting “most recent” in PubMed, the range bar in IEEE Xplore and the setting “since 2010” in other databases, such as Scholar to highlight articles from the last ten years (from 2010 to 2020). The reason is to have insight on the most recent advances in methods for respiratory assessment. The results were added to Mendeley database. The selection process involved the following inclusion/exclusion criteria:

- The paper should be published as a journal article or in conference proceedings.
- The paper should be written in English.
- The paper should aim to characterize or quantify a respiratory element.
- The paper should be based on non-contact systems to assess respiration, especially the camera-based systems.
- Other vital signs, such as heart rate quantification, are not included in this review.
- If a paper from a specific research group or project had been published in a conference and then in a journal, only the extended journal paper was reviewed.

Most of the papers present methodological studies, which are conducted to develop, experiment, and validate new methods, techniques, and systems to assess the respiration. In our inclusion criteria, we focus on works characterizing or quantifying one or more respiratory elements using innovative ways. The description of each element is given in Appendix A. This includes respiratory rate (Appendix A.1), volumes (Appendix A.2), blood gas concentrations (Appendix A.3), and chest wall movement, patterns, or deformities (Appendix A.4). Moreover, a particular emphasis was given, in this work, to the non-contact aspects, methods, systems and their applications through the retrieved works, especially the camera-based approaches.

**Figure 2:**
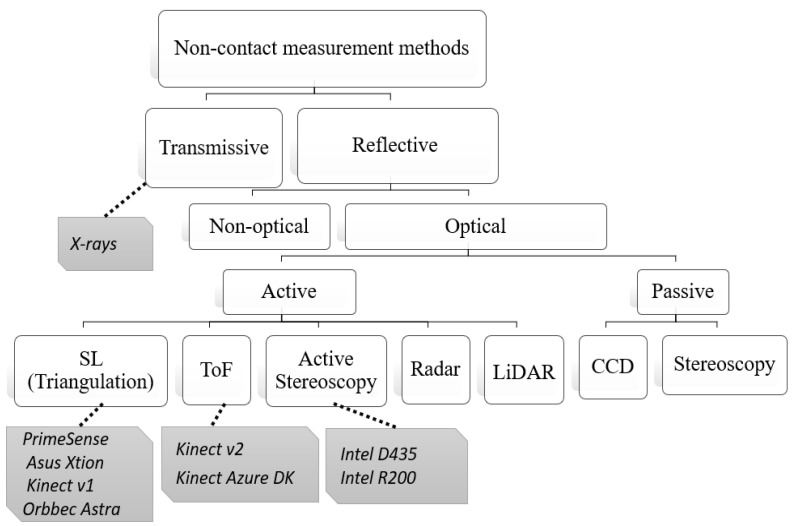
Non-contact sensors’ technology classification: SL = Structured-Light, ToF = Time-of-Flight, LiDAR = Light Detection and Ranging, CCD = charge coupled device.

Non-contact methods are also called remote, contactless, wireless, or contact-free methods because of the absence of contact with the subject. In this category, devices are not attached to the body. Main contactless sensors can be categorized into radio frequency-based and imaging-based systems. Imaging devices include RGB video, thermal, ultrasound, and depth sensors. These devices differ primarily in their data types and technologies, as shown in Figure 2. Some theory related to non-contact technologies is described in Appendix B. This includes radars (Appendix B.1), cross-sectional, radiography, and fluoroscopy imaging sensors (Appendix B.2), RGB, and thermal sensors (Appendix B.3) and depth sensors (Appendix B.4), which are based on Structured-Light (SL), Time-of-flight (ToF), and Active Stereoscopic Vision technologies (ASV).

While they are considered as relatively new and not fully mature, many non-contact methods have achieved good outcomes through a few works. The results are given in Section 3, along with a comprehensive description of the reported methods.

## 3. Results

**Figure 3:**
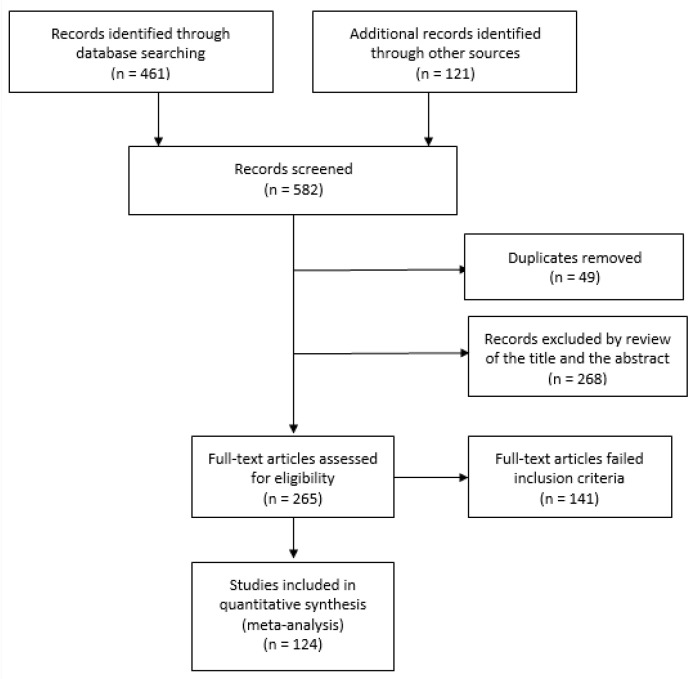
The flow diagram for studies included according to PRISMA.

Our initial search identified a total of 582 references. As illustrated in the flow diagram of Figure 3, 54.46% of the retrieved papers (n = 317) were eliminated after duplicates removal, title, and abstract screening.

Most excluded publications either required direct and close contact while measuring the respiratory parameter or quantified other vital signs (e.g., heart rate). A total of 265 publications were included in the full text review, during which we excluded 141 publications based on the fixed inclusion/exclusion criteria. Data were extracted from the remaining 124 publications.

**Figure 4:**
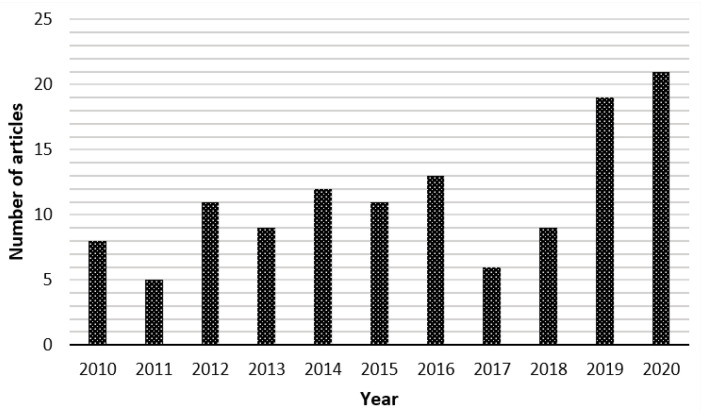
Number of articles per year. Only articles published prior to 31 October 2020 are counted.

Figure 4 shows the number of retrieved papers per year in the period between 2010 and 2020. We observe that many papers were published during the last five years (more than 70% of the selected articles). Many groups conducted considerable research in this area, which provided valuable results. This is due, as well, to the emergence of camera technologies, especially the depth sensors. Many companies, such as Microsoft and Intel, have produced a variety of low-cost commercial cameras. Looking more closely into each article, we notice that most works are addressing the respiratory rate (48%) and the chest wall motion (37%). Some works addressed more than one respiratory element in the same paper (e.g., respiratory rate and tidal volume or respiratory rate and chest wall motion, etc.).

Non-contact hardware system development brought many new innovative methods, some of which were less efficient or even impossible to use in the past. Most of the retrieved papers included a methodological section explaining the proposed technique, followed by experiments and the validation sections. Experiments are aimed to investigate the applicability of the method with supporting data and analyze it appropriately. Validations are aimed to provide convincing proofs regarding the ability of proposed systems to approach the accuracy of conventional gold standard methods. Many works used signal processing and computer vision techniques, such as filtering, optical flow (using Horn–Schunck algorithm), thresholding, segmentation, principal component analysis (PCA), image analysis, surface reconstruction, etc. Table 1 shows a comprehensive view of some of the non-contact techniques, along with their results between 2017 and 2020.

Respiratory rate seems to be one the most attractive parameters using the non-contact methods and has been addressed in a variety of conferences and journal papers divided as follows: [19,56,57,58,59,60,61,62,63,64,65,66,67,68,69,70,71,72,73,74,75,76,77,78,79] (over the period 2010–2015), [9,11,12,29,32,42,44,45,46,47,48,49,50,51,53,55,61,69,80,81,82,83,84,85,86,87,88,89,90,91,92,93,94,95,96,97,98,99,100,101,102,103,104,105,106,107,108,109,110,111,112,113,114,115] (over the period 2016–2020).

Monitoring of pulmonary function such as tidal volume, minute ventilation, forced vital capacity, and forced expiratory volume has been studied in many works as well in [65,116,117,118,119,120] (2010–2015), [6,7,8,9,32,46,52,88,91,92,100,103,110,121,122,123,124] (2016–2020).

Blood gas concentrations (oxygen saturation and end-tidal carbon dioxide monitoring) has been addressed in [15,80,125,126,127].

The characterization of chest wall motion has been addressed in many methodological works, which presented interesting results, including for the general (e.g., motion quantification) and specific levels (e.g., illness detection/severity assessment and scoring). This can be grouped as follows: [57,61,62,64,68,72,117,120,128,129,130,131,132,133,134,135,136,137,138,139,140,141,142,143,144,145,146,147,148,149] (2010–2015), [3,10,47,48,49,53,54,95,97,99,101,105,111,122,150,151,152,153,154,155,156,157,158,159,160] (2016–2020).

The respiratory rate, ventilation, gases concentration, and chest wall motion were measured in a variety of subjects, including healthy volunteers, newborns, infants, very ill patients, and elderly people. Studies on simulated data are reported as well in some of the selected research papers.

Methods used to characterize and quantify these respiration elements were based on the following non-contact techniques: radar detection, cross-sectional sensing, ultrasonic technology, radiography, fluoroscopy imaging, RGB conventional imaging, thermal imaging, and depth sensing, which involves the structured light, time-of-flight, and active stereoscopic vision technologies. An overview of these technologies is given in Appendix B. The remainder of this section provides a description of different methods and organizes them based on the technology similarity.

### 3.1. Radar Sensors

Reference respiratory rates ranges were extracted from a review of observational studies that used respiratory rates data from 3881 children (from 6 months to 18 years old) [161]. Based on 99th and 1st centiles for children and young adults, the RR could range from 8 to 60 breaths/min (0.14 to 1 Hz, respectively. The range in adults is much more restricted but would be included in this range. An extreme range may occur in critical illness, such as an elevated RR (>40 breaths/min in children with pneumonia) as an early indicator of critical illness. Therefore, the maximum value peak frequencies in the respiratory frequency band (0.14 to 1 Hz) were automatically extracted, reflecting RR.

One of the first monitoring techniques, radar-based, was described in 1997 by Greneker et al. [162]. The system was called the Radar Vital Signs Monitor (RVSM). It was proposed to examine the performance of Olympic athletes at distances more than 10 m. The RVSM calculated breathing-induced movements of the chest based on the Doppler physical phenomenon. A big limitation of the RVSM is the observed motion artefacts, which degrades the respiratory signals. There have been no available studies illustrating the testing of this technique in infants.

Use of continuous wave (CW) unmodulated microwave Doppler RADAR at 2.4 GHz and 1.6 GHz was reported in the context of a thesis work by Droitcour et al. [163]. By analyzing the shift in frequency, authors estimated the motion of the thoracoabdominal wall. RR was detected from a range of 1.5 m, from a population of 22 patients. The work was validated using gold standard based-contact systems (thoracic impedance and inductive plethysmography).

In the last few years, a variety of radar-based sensors has been investigated for respiration monitoring [44,51,69,82,94,95,96,97]. In 2019, Nosrati et al. [94] proposed a phased array approach with a multiple-input multiple-output (MIMO) beamforming design. The system can detect multiple targets and hence offers a good accuracy for short-range human vital signs monitoring. Another radar-approach for respiratory assessment was proposed by Adib et al. [69]. Authors used a frequency modulated continuous wave (FMCW) in the range of 5.46 GHz to 7.25 GHz, rather than using the Doppler effect. The subject was in another room then the radar’s room and his RR was accurately measured with a working distance of 8 m.

A potential application of this system is to monitor patients with transmissible infectious diseases, such as the recent severe acute respiratory syndrome-associated coronavirus 2 (SARS-CoV-2). An important requirement to help caregivers to identify potential infected patients is to detect those with abnormal breathing using remote sensing from over 2 m away. This radar-based FMCW technique can be used as well in the context of “smart homes” by incorporating the sensor in a Wi-Fi router for RR monitoring in a residency given its wide sensing range capabilities [69].

Table 2 describes the latest works, particularly those of 2020, using different radar architecture such as FMCW and pulsed Doppler radars.

Despite this increasing interest of research works in radar-based technologies, the current technology of radar-based techniques has several major shortcomings. Body movement interferences [104,164] and the lack of efficient and stable signal processing techniques, which would be capable of handling low samples of data [70], are two examples of these issues.

### 3.2. Cross-Sectional Imaging Sensors

Computerized tomography (CT) has been used in many works on chest wall assessment. However, techniques based on CT scans may suffer from motion artifact. Therefore, PCA has been used to decrease the number of artifacts and to clean CT images [165]. Another interesting technique using 4D-CT images acquired in cine mode to model respiratory motion is proposed by Yan et al., applying optical flow (OF)-based deformable registration [166].

Magnetic resonance imaging (MRI) is another cross-sectional imaging technique, helping to achieve better characterization of chest wall motion. Since MRI incurs lesser ionization then CT scanning, it has been used in a number of studies [165,166,167,168,169,170]. Interesting and promising results have been achieved in regional chest wall motion assessment using MRI imaging, including chest wall behaviors during breathing in pectus excavatum [171] and scoliosis [172]. Due to their reliable and high performance, 4D and cine-MRI images have been used in numerous works, estimating organ or tumor motion resulting from respiration [173,174]. However, MRI needs expensive technology that cannot be used outside a dedicated radiology unit.

### 3.3. Ultrasound Imaging Sensors

For instance, Liu et al. [175] employed an ultrasound system to evaluate the relationship between the diaphragmatic excursion distance and tidal volume. Their system uses a spirometer and an ultrasound probe to collect data while a volunteer performs a breathing session. The diaphragm area is detected in each ultrasound image frame using a histogram of oriented gradient (HOG) descriptors [176]. The diaphragmatic excursion distance is calculated by tracking the diaphragm contraction and relaxation. Spirometer data are used as ground truth to find a relationship with the diaphragm excursion distance. The results show that there is a linear regression relationship between diaphragm excursion and tidal volume. This relationship is then used to estimate tidal volume when conducting ultrasound examination.

While these experiments yielded good results, the authors reported possible failures in diaphragm detection. These failures may be caused by diaphragm image incompleteness or by the detection of a tissue or of an organ that has a shape similar to that of the diaphragm. Furthermore, the probe location and its angle significantly influenced the average error between the spirometer record and the predicted tidal volume. Thus, it is an operator-dependent method.

Similar, but more elaborate techniques have been proposed using ultrasound transmission [177] and laser displacement sensors to measure ribcage and abdomen movements [178]. In ultrasound-based techniques, transmitters positioned on the thoracoabdominal zones transmit signals to an external receiver to evaluate linear movements at various regions of the thorax and the abdomen.

Lafortuna et al. [179] proposed an alternative method measuring the delay between the emission and reception of ultrasound signals at opposite ends of two rubber tubes surrounding both ribcage and abdominal areas.

Although most ultrasound techniques showed interesting results in calculating respiration parameters, such as volume, it has not been tested for infants and children.

### 3.4. Radiography and Fluoroscopy Imaging Sensors

A major shortcoming of approaches based on radiography and fluoroscopy imaging is that they present higher cost, slow acquisition, low resolution, and more noise than other imagining techniques, such as digital imaging, and especially patient exposure to an extra dose of radiation [145,165,180]. Moreover, some of these systems also have the problem of dependence on fiducial markers, making for complex and slow preparations, which may be extremely disruptive to the patient, and increasing the treatment time. Additionally, the constraint of having to stay inside the CT/MRI device during the examination restricts these systems and make their application complicated with children, newborns, elderly people, and patients with acute diseases. Thus, these approaches cannot always be applied in pediatric intensive care or other specialized environments.

### 3.5. RGB Sensors

Many techniques have been proposed for respiration assessment, using RGB sensors, such as classical webcams [72].

Researchers have used image processing techniques such as optical flow [181,182], image subtraction [79], and remote photoplethysmography [29], which consists of illuminating the skin and then measuring the changes in light absorption.

For instance, Kumar et al. estimates the breathing rate through recording the skin color variation (specifically near the lips). The variation was recorded with an RGB camera [71]. This technique passes through a judicious selection of the region of interest. This technique is sensitive to skin color, and results may be skewed by ambient lighting, etc. A number of research works have been proposed to overcome these limitations using signal processing and statistical modeling techniques, such as blind source separation, alternative reflectance patterns, spatial pruning, and temporal filtering [58,75,183,184].

Tan et al. [57] developed a system for respiratory rate estimation. Using a single video camera, the system uses a 2D image subtraction technique to detect the repetitive movements of the chest and abdomen caused by breathing. The respiratory rate results were evaluated against a thermistor, a stain gauge, and a flow monitoring system, but only subjective assessments were reported [6].

Ying-Wen Bai et al. [79] used double video cameras and a temporal differencing algorithm to calculate the breathing rate. By detecting moving objects between 2D frames, the system monitors and records the patient’s breathing signal. Their algorithm also detects the chest expansion and contraction, which allows the respiratory rate to be easily deduced.

Benetazzo et al. measured breathing rates using a weighted averaging filter applied to a region of interest on the chest wall [75]. While the results were good, the system expects the user to be sitting in a frontal position with an angular orientation not exceeding 25°. The region of interest (ROI) is segmented by using the camera skeleton’s joint information. Their results were validated using a spirometer, with an outcome of 0.98 correlation. One drawback of this technique is that it cannot be extended to calculate other respiratory parameters, such as airflow or tidal volume due to its very limited spatial coverage.

Alinovi et al. used a video processing-based system to estimate the respiratory rate [56]. Their computer vision approach combines two recently presented techniques. The first technique amplifies small movements that are difficult to observe with the naked eye, such as the respiratory movements present in a video stream, detecting them by applying a spatial decomposition and temporal filtering [138]. The second technique estimates a frequency from many signals (using many cameras simultaneously) [73]. This approach performs a multi-resolution decomposition to the video frames, transforming the original frame into a pyramidal representation with different spatial scales. This representation is then temporally filtered pixelwise using the infinite impulse response (IIR) filter. This same filter is used to extract the components whose periodicity is compatible with the breathing rate. A single motion signal is then calculated at each level. The breathing rate is deduced from the extracted signals by applying the maximum likelihood (ML) criterion. This system was validated against a gold-standard polysomnographic system and showed a good agreement in respiratory rate estimation. Although the results indicate that the proposed method handles small breathing movements adequately, the method does present some potential weaknesses. The researchers used a gold standard (plethysmography) presenting a high degree of uncertainty when the patient is moving (e.g., newborn babies). This method also has a high level of complexity. In addition, the accuracy of this assessment depends on the type of temporal filter utilized. The IIR filter these researchers selected only extracts periodic variations [73]; thus, would not be appropriate to extract the breathing motions of severely distressed patients in pediatric intensive care unit (PICUs), as they may not be periodic.

Frigola et al. [185] developed an algorithm to continuously monitor a patient and detect any potential apnea that may occur during sleep. The algorithm monitors inhalation and exhalation by detecting body movement using optical flow. For validation, the authors used an elastic cloth band as their gold standard method, but the comparative evaluation results were not reported [6].

The trend is moving towards using embedded devices’ cameras, such as in smartphones and tablets. Indeed, these intelligent devices present higher resolution improved over time, and are some of the most important devices available today for consumers. A contactless method, based on a smartphone camera, has been developed by Reyes et al. [100]. Breathing rate and the tidal volume were calculated from intensity variations in the video channels. These variations are generated from chest wall expansion and contraction during breathing. The authors utilized a spirometer as a gold standard system to record the tidal volume and to validate their procedure. The smartphone and spirometer breathing patterns were identical as evidenced by the high values for the coefficients of determination ($r^{2}$ = 0.95). Despite these optimistic results, the results may be influenced under varying lighting conditions.

One of the disadvantages of RGB sensors is the lack of accuracy when tracking the movement of respiration. To address this issue, many works were based on tracking fiducial markers to improve tracking accuracy. For instance, Wisner et al. [186] placed color fiducials on the patient’s abdomen to track their motion. Authors used an RGB color webcam and computer vision techniques, such as thresholding and PCA to produce 2D color images. To validate their technique, the authors used a system providing ultrasound images. Results show a high correlation between the signals from both techniques. The main disadvantage is that placing markers on the patient could disturb the doctors during their intervention may be the patient as well. Besides, markers can be displaced from their initial locations during the subject’s movement.

### 3.6. Thermal Sensors

Measures of thermal changes have been used to monitor respiration rate with infrared (IR) video [59,65,187,188,189,190,191,192,193]. Wang et al. present a real-time IR imaging system for identifying abnormal breathing and describe its clinical application in the detection of obstructive sleep apnea episodes during sleep.

Hsu and Chow [193] developed a thermal sensor-based respiration rate monitoring system designed for use on children. This approach does not require any contact with a child’s skin. Instead, the sensor detects temperature variations due to breathing and then corrects and analyzes the data simultaneously using a processor linked to a principal nursery room. To not lose the respiratory signals, authors have placed many thermo sensors in an ellipsoid-shaped mask. The aim is to estimate the respiration even if the subject turns his head. The constraint of placing the mask near to the subject’s face was the main drawback of this technique.

A computer vision method for respiratory rate estimation was proposed by Zhu et al. [194] using an infrared camera. Authors designed a tracking system following the facial features and then deducted the breathing rate. These features were manually selected from a reference image (i.e., the first image in the video) by identifying three windows. Two of them cover the areas between the bridge of the nose and the inner corner of the eyes (i.e., the periorbital regions). This corresponds to the warmest areas of the face. The third window is positioned over the apex of the nose to characterize the coolest facial area. These three windows were tracked in the successive frames. The respiration signal is estimated from a rectangular zone under the nose.

Other works measured the breathing rate by measuring the temperature variations around the neck region, the carotid vessel complex, and the nasal region [188]. In their work, the authors utilized a long-wave thermal camera consisting of a focal plane array for an infrared (6–15 µm) sensor. The FLIR A40 thermal camera was used to record the skin surface temperature variation in a region centered on the tip of the nose [189]. This sensor was presenting a good thermal sensitivity of 0.08 Kelvin and a high frame rate of 50 images per second. In the experiment, the respiration of a patient was recorded over two minutes. The sensor was fixed on a support in front of the subject at about one meter. The images were acquired, segmented, and processed by tracking a circular area centered on the tip of the nose. The tracked area is split into eight equal concentric segments. The skin temperature of the segment is calculated through averaging the pixels values in the segment. By calculating skin temperatures in each frame, authors compute plots of temperature over time. The breathing rate was deducted from the calculated plots.

### 3.7. Depth Sensors

One of the most insightful and complete comparisons of commercial depth cameras was proposed by Giancola et al. in [195]. Authors compared twenty commercial depth cameras and their technologies in indoor environments, such as Kinect v2 (ToF), Orbbec Astra (SL), and Intel D435 (ASV). They demonstrated that the uncertainty in the depth sensing using a Time-Of-Flight camera, such as Kinect v2 scales linearly with the depth, thus giving accurate measures at longer ranges. Structured-light based sensors give measures with uncertainty increasing quadratically. Giancola et al. concluded that structured light-based sensors are preferred in short-range applications. The work, however, did not provide examples of the application of depth sensors in the medical field and specifically for respiration applications.

Respiration assessment were explored through a few works based on commercial RGB-D sensors for color and depth images acquisition. Computer vision and graphics techniques have been applied for data processing for both color and depth images, such as image filtering [56,63,72,138], image subtraction [79], principal component analysis models formed from images to describe breathing variation [146], averaging depth values in thorax regions from depth images [141], estimating statistical modes of motion field variation during respiration [143], or by fitting existing motion models [33], and using Iterative Closest Point algorithms to improve surface reconstruction techniques [132,140,196].

#### 3.7.1. Structured-Light (SL) Sensors

Several works investigated [67,77,78,116,129,134,135,141,151,158] the performance of the commercial SL sensors in respiration assessment. For instance, Noonan tracked the respiratory signal waveform by computing depth maps from a single SL camera [134].

Martinez et al. [78] projected a few dots on a patient’s chest and tracked them using their SL camera’s IR sensor for over 30 s. Their method then filtered trajectories using principal component analysis (PCA) and calculated the respiratory rate using autoregressive spectral analysis. The main drawback of this work is the limited spatial coverage since authors were based on a specific number of points and not the entire surface. Thus, the method cannot be extended to extract pulmonary function parameters.

Bernacchia et al. proposed a measurement method for the monitoring of heart rate and respiratory rate in healthy subjects at home [67]. Breathing activity was measured simultaneously with a spirometer on a time window of 30 s. While their results show good correlation with the spirometer, their method suffers from low spatial coverage since it only allows a part of the abdominal area to be covered.

In [135], Xia and Siochi used only depth images to calculate the average depth over a thoracoabdominal area, manually extracted by positioning a translation surface over the image. However, both works [134,135] are basic feasibility studies of how to compute breathing motion waveforms from depth maps and do not allow a concrete assessment of respiration by quantifying breathing parameters, such as respiratory rate and tidal volume.

Burba et al. [77] defined the chest cavity as a rectangular region, outlined using the joint positions in the skeletal model provided by the sensor software development kit (SDK). Only the top half of the torso was taken into consideration to characterize the displacement due to breathing motions. The subject movement may, however, affect the method’s accuracy.

Ostadabbas et al. [118] used a single SL camera to calculate two respiratory volumes: the forced volume vital capacity and the forced expiratory volume of air one second after full inspiration, and then deduced the airway resistance. The chest was defined as a rectangular region of interest bounded by the following points: “right shoulder”, “left shoulder”, “right hip”, and “left hip”. These points were calculated from the skeletal information in the SDK camera. They then estimated the lung volume by numerically integrating the depth value in the ROI. Their approach showed a good agreement with a standard spirometry test, with a 95% confidence interval and an average 0.88 correlation between the volume/flow estimations measured by their method and those measured by a spirometer.

Yu et al. [116] developed a system to estimate tidal respiratory volume by deducting it from the calculated length per pixel and the depth map acquired from a single SL camera. They designed their system to accommodate users in a sitting position at 1.4 m in front of the RGB-D sensor. Three regions of interest were defined at three specific positions (left thorax, right thorax, and abdomen). A predefined chest wall mask must be adjusted (position and size) to fit the patient’s thoracoabdominal region. The regional morphological changes in the chest wall are then directly calculated from the depth image. The experiment was conducted indoors and reported an overall correlation of 0.96 against a standard spirometer. However, the authors reported that the estimated respiratory volumes of the system were less than those obtained from the spirometer, due to the low spatial coverage of the regions involved in respiration. Their system does not measure the morphological changes in the side region of the chest wall.

Seppanen et al. [129] followed the chest wall breathing airflow patterns using a depth camera based on SL technology. Their results were endorsed by those of a spirometer. Authors reported a high value of the coefficient of determination between the two signals ($r^{2}$ = 0.93).

The respiratory motion data variance was analyzed by Tahavori et al. [141] using a single SL depth sensor. The camera was positioned on the top of the chest, then on the top of the abdomen, to estimate the average depth value of sixteen regions of interest on the thorax and the abdomen. By applying the PCA algorithm, authors found that more than seventy percent of the motion data variance of the thoracoabdominal surface are indicated in the first component.

The respiratory systems using a single camera such as in [116], may suffer from a limited spatial coverage. To address these limitations, a number of works used multiple SL cameras for surface reconstruction. For instance, Harte et al. [151] used four RGB-D sensors to analyze the thoracoabdominal small amplitude movements, corresponding to the breathing activity. The sensors were positioned around the patient at 1 m to reconstruct the patient’s thoracoabdominal area. The volume changes obtained by this technique were compared to those obtained by a spirometer. The experiments have yielded good correlation between the proposed system and the spirometer in volume estimation. Nonetheless, the system was unsuitable to fit the clinical environment because of the system’s complex settings and the big number of used sensors [91]. Moreover, the system measures were not correctly synchronized in time and frequency, yielding a number of errors in the 3D reconstruction [158]. A system using two Kinect v1 sensors has been developed by Heß et al. [148] for respiratory gating in a positron emission tomography (PET) study. The aim of using more than one camera is to enlarge the spatial coverage of the respiration zone. Authors validated their procedure by using a moving high-precision platform. The platform position was calculated with a small mean margin of error of 0.2 ± 0.11 mm at a 75-cm measurement distance, and 1.27 ± 0.30 mm at 125 cm. Additional experiments involving 10 healthy subjects and 10 cancer patients showed that abdominal signals were more suited for PET gating data than the thoracic signals.

#### 3.7.2. Time-of-Flight (ToF) Sensors

ToF sensors, such as Kinect v2, have been explored for close range sensing in many recent works, such as in [39,197]. The Kinect v2 ToF sensor does not only provide higher resolution RGB image than its previous SL version (Kinect v1), but it also yields more accurate and denser depth measurements [39]. It has been shown that Kinect v2 depth resolution are 2 mm under 3 meters’ distance [197]. Moreover, it has lower axial and lateral noise in the acquired depth information than the SL Kinect v1. The Kinect v2 sensor is able to operate under a variety of conditions, such as performing measures under shadow and direct sunlight exposition and even under significant near infrared (NIR) interference from halogen lamps or sunlight. [39,198,199,200,201,202]. Moreover, the systematic and non-systematic errors of depth measurements are reduced in Kinect v2 compared to its SL predecessor. Despite this, both Kinect SL and ToF versions, present advantages, and shortcomings, depending on the task to be performed [36].

The Kinect v2 sensor has been compared with another SL sensor in [203] (the Carmine 1.08). Authors show that the Kinect v2 presents better depth precision and angular resolution. Another comparison between the Kinect v2 and the SL Asus Xtion Pro comes to similar conclusions [204]. In Addition, the Kinect v2 provides more accurate depth data for both indoor and outdoor use.

Other works investigated the performance of different commercial ToF sensors in respiration assessment [91,131,205,206,207]. Penne et al. made one of the first attempts to compute breathing pattern using a low-cost commercial depth camera based on time-of-flight technology [205]. In their method, authors were calculating the best-fitting planes for the thoracoabdominal area. Then, breathing patterns were deducted by tracking the displacement of each plane relative to a reference plane corresponding the subject’s bed. Finally, the authors validate their system against an ANZAI belt system (AZ-733V, ANZAI Medical Co Ltd., Shinagawa City, Tokyo, Japan,), by evaluating the correlation between the quantitative measures. The experiments were performed by positioning the belt on the thorax first, and then on the abdomen. The analyses revealed a correlation of 0.85 (in the thoracic region) and 0.91 (in the abdominal region) between the two systems.

Falie et al. proposed a system for apnea detection, by recording the thoracoabdominal movements during sleep [206]. By using a ToF camera (SR3000 sensor model), the authors divided the thoracoabdominal zone into 12 regions, in which they distinctly evaluated the motion. The aim was to monitor the subject’s respiration and detect any irregularity, which may be associated with potential sleep apnea. Schaller et al. utilized the same camera model (SR3000) to compute the real time respiratory pattern without markers [207]. The authors reported that it is feasible to acquire a 3D model in real time using a single camera and to simultaneously compute the thorax and abdomen breathing motion. They note that it is possible to split the chest wall into many regions and estimate those regions’ relative breathing patterns using their methods. To validate their technique, they acquired the thoracic and abdominal breathing patterns of 13 (healthy) volunteers using an external gating system, the ANZAI belt. The two system measures were highly correlated. Ulrich et al. developed a novel anatomic-like mannequin to reproduce the thoracoabdominal breathing movements [131]. The aim was to handle moving tumors in the thorax and in the abdomen during radiotherapy, which can be by irradiated through respiratory gating. The phantom was designed to simulate the thorax and abdominal motion for surface-based respiratory gating systems. They used the ToF technology for chest wall assessment. The breathing pattern of the phantom was already known. The correlation between the gold-standard signal performed by the phantom and the estimated pattern by the ToF sensor is 0.65 for respiratory amplitude of 1.5 mm and above 0.80 for amplitudes greater than 5 mm. In addition, authors reported that the designed system can detect a frequency of at least 25 respiratory cycles per minute. The phantom is a promising practice tool to handle the tumor or organ motion resulting from breathing motion.

In a study carried out by our group [91], two optical commercial ToF cameras were used to calculate respiratory rate and tidal volume for age group categories ranging between birth and 18 years. The respiratory rate was derived from the dynamic volume calculation between consecutive frames. This method was tested in an environment designed for critically ill children, where it was compared to the mechanical ventilator, a gold standard method used in intensive care units.

**Figure 5:**
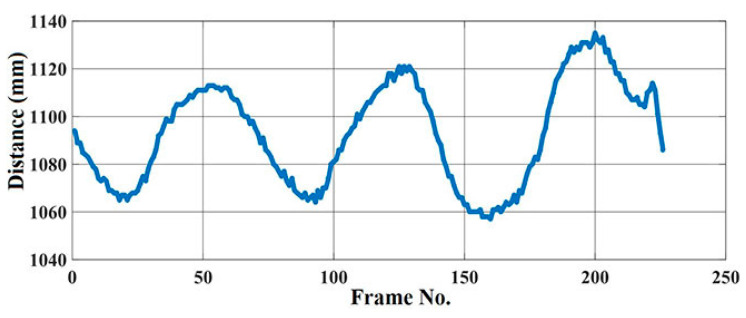
Depth variation over 8 s using the Kinect v2 ToF sensor (camera frequency: 30 frames per second).

In Figure 5, we conduct an experiment using a ToF Kinect v2 to calculate the average depth variation from a rectangular torso region of an artificial test lung simulator (MAQUET Medical Systems, 1 Liter Test Lung 190). The respiratory rate can be directly derived from these variations.

#### 3.7.3. Active Stereo Vision (ASV) Sensors

The performance of ASV depth cameras in capturing depth data indoors and outdoors, have been investigated through a number of works [37,208]. Keselman et al. evaluated the Intel R200 ASV sensor for indoor environments. Authors concluded that the R200 is appropriate for lower-tolerance 3D applications. The R200 can acquire data in outdoor environments as well, unlike the traditional SL sensors.

In [209], authors compared, through an experimental study, the three technologies using one Asus Xtion Pro (SL), one Kinect v2 (ToF) and finally one Intel R200 (ASV) for indoor 3D reconstruction. The performed experiments yielded higher performance in Kinect v2 ToF sensor with less noisy and denser 3D information. Another comparison in [210] was performed based on two SL sensors (Kinect v1, Asus Xtion Pro), one ToF (Kinect v2), and one ASV sensor (Intel R200). The comparison yielded to the same results and conclusions as [209], and showed that the Kinect v2 ToF sensor surpasses the rest of the three devices and presents higher spatial resolution

The number of works based on ASV sensors for respiration assessment is relatively limited. The reason is that the commercial ASV sensors are very recent. The R200 sensor (2015) was first in the Intel ASV family, which includes D415 and D435 (2018) as well. Another reason is that the Kinect family were the first and most used commercial RGB-D sensors in respiration assessment, not only for their dense acquired depth data, but also for the Microsoft rich SDK functions.

Schätz et al. used a variety RGB-D sensor, including the D415, and D435 ASV sensors to capture the breathing rate of a sleeping person [111]. Authors showed that depth sensors could be used to record breathing rate with the same accuracy as contact sensors used in polysomnography (PSG). They also showed that signals obtained from the depth sensors have the same sensitivity to breathing changes as in PSG records. Table 3 shows the properties of the D415, and D435 ASV sensors, among different types of recent depth sensors.

A hybrid radar camera prototype was proposed in [109], to monitor the respiration of multiple subjects, simultaneously. The system involves an ASV D415 sensor working jointly with a low-cost impulse-radio ultra-wideband (IR-UWB) radar to detect the person location and then extract his respiratory rate information.

## 4. Discussion

In this review, we provide a synthesis on non-contact technologies for respiration assessment and characterization. The results of a variety of methods based on non-contact technologies were illustrated in the previous section. We grouped the articles based on different non-contact technologies. We want to explore what the applications are of these works, in what area they can be used, and how far we can go. We believe that the answer is best understood in terms of answers to these three questions:

- In which operating environments do non-contact systems perform?
- What are the limitations on what can be achieved in respiration assessment, using non-contact systems?
- How can non-contact systems help address some of the current and urgent health issues in the present year (2020)?

Below follows a discussion on these three questions in relation to prior and current research.

### 4.1. In Which Operating Environments Do Non-Contact Systems Perform?

Despite the availability of many non-contact systems for respiration monitoring, they are all still far from being in broad commercial use. These systems are especially sensitive to artifacts of various origin, such as patient, background movement, and ambient light changes. Thus, they cannot be used without the guidance of qualified personnel. In addition, some environments are more demanding than others. Thus, a knowledge of the environment is very important and may directly affect the effective application of the respiratory system. We discuss some requirements related to each environment. There is no universal solution to work in all environments; however, the ultimate respiratory monitor would provide continuous information about respiratory parameters without causing any sort of disturbance to the subject. That system should accommodate the environment, fitting its conditions and requirements. A mobile respiratory system, for instance, would be easy to use in various environments, such as in transports (on airplanes and/or in trains in case of an emergency).

Standardization and facility of use will allow anyone to measure respiratory parameters and send the data to a health professional. The current proposed solutions are still far from being in wide use in the different environments due to their many constraints, such as a system’s complexity and ease of use, space occupation, the need for connection to computers for data processing, system mobility, and the presence of a trained health professional, as well as other constrains related to each environment. Some environments are overly strict and more demanding than other environments.

Table 4 summarizes recent respiratory systems that have potential ability to perform in one or more of real-life environments. Their applications are addressing problems associated to respiration such as general health assessment, respiratory symptoms or diseases detection, sleep assessment, athletic performance monitoring, lung conditions monitoring, driver drowsiness detection, etc.

Table 5 shows the tolerance levels for the main common constraints related to the different environments. For example, authors in [91] address respiratory parameter assessment in a pediatric intensive care unit (PICU), in which the different services are affected by complex settings and unexpected urgent interventions. PICU rooms are not big enough for their designated daily procedures, devices, and the number of infants/children. It is extremely crucial to fully control the free space. Any occupied area should not cause disruptions. Authors also address emergency cases. The proposed system can effortlessly and rapidly separate/disconnect from the bed permitting the transportation of the subject.

In this section, we give some examples of works that addressed the following environments: home, clinical, sports, cars, and intensive care. Other types of applications are briefly described, such as real-time detection of suicide attempts and respiration assessment in aircrafts.

#### 4.1.1. Home

Respiratory systems devices can now be used at home for a variety of applications (Table 4). For instance, the general state of health of the newborns (good sleep quality, calm behavior, etc.) can be monitored using the depth cameras. By monitoring a baby’s sleep, the cameras-based systems may help to prevent sudden infant death syndrome (SIDS) in children of less than one year of age. Other applications include the detection of respiratory illness (asthma, obstructive sleep apnea, etc.). In chronic obstructive pulmonary disease (COPD) patients, lung conditions may be monitored using non-contact systems. Non-contact systems may be used as an aid in reducing anxiety states, as well, by controlling the respiration rate. This activity is known as respiratory training.

In 2016, Procházka et al. [93] have suggested a structured light camera-based system to monitor the respiratory rate. The authors claimed that there is no significant difference between biomedical features described by different biosensors and the non-contact depth sensor. The system can be used at home to monitor the general health assessment or even to analyze physical activity of elderly people to facilitate performing some tasks in old age.

#### 4.1.2. Clinical

In clinical practice, a variety of diagnoses are based on respiratory assessment as early indicator of severe illness or to evaluate the progression of illness. The applications range from general health assessment to the management of respiratory motion in PET/computed tomography.

Sharp et al. [122], for instance, conceived and implemented a breathing system for thoracoabdominal three-dimensional movement tracing and lung volume assessment. The system can be employed to examine breathing rate without contact with the patient, screen for abnormal spirometry and to support ventilators in quantitative assessment of respiration. Sharp et al. mention that the system also has the potential to monitor regional thoracoabdominal motion in more detail (e.g., for the detection of pleural pathology, such as effusion and pneumothoraces). The system can be used in both hospital and home settings. However, the authors specify that patients were installed upright with their arms by their sides on a standard chair with no armrests, facing an RGB-D sensor placed at 1.5 m from the subject, at a height of 0.6 m. Patients were afforded with figure-hugging t-shirts to increase the precision of the thoracoabdominal motion evaluation. These conditions are not suitable in real clinical environments. Even though this technique in not expensive and does not require specialist equipment and training as with spirometry, the conditions applied are still rather strict and do not accommodate many categories of population, such as elderly people, very ill patients, infants, and newborns.

#### 4.1.3. Sports

Respiration has always been closely linked in athletic training and performance improvement. Camera-based systems can be used to help athletes control their breathing without a visual guide, by tracking and measuring their breathing during training.

Aoki et al. [103] developed a non-contact system for respiration measurement during bicycle pedaling exercise using a Kinect sensor facing the test subject. The authors used an incremental extraction technique starting from the detection of the human body using depth data to the extraction of the region where the respiration component is more dominant. Next, they extract the pedaling motion component using a Fast Fourier Transform (FFT) band pass filter. Finally, the respiration waveform is calculated by computing the volume change in the respiration region.

The system is very promising in environments, such as sports clubs. However, it presents some substantial drawbacks. The authors assume that the athlete is always in a sitting position. However, the athlete may change his or her position during pedaling (standing, sitting, and otherwise moving their body). Moreover, the system is only valid for a bicycle ergometer. The size of the Kinect sensor may be inappropriate for a standard real bicycle. Furthermore, the system is attached to a computer for processing. Indeed, it cannot be tested on a real bicycle. One possible way to improve this system would be to replace the computer by an embedded processing system.

#### 4.1.4. Cars

Detecting drowsiness while driving is an important topic of research. Several research studies attempt to detect the state of distraction while driving [211], by means of electrocardiogram (ECG) [212] or electroencephalogram (EEG)-based brain-computer interfaces (BCI) [213]. The analysis of respiration regularity is one of main techniques used to detect the driver’s drowsiness or impaired driving [66,106,214]. For instance, detecting the attempts of fight against falling sleeping while driving has been investigated in [106]. The proposed system evaluates the respiratory rate variability to detect the fatigue patterns.

Ripoll et al. [98] proposed an on-board camera system for the detection of drowsiness. The system performs analyses of the driver’s physiological parameters, such as the respiratory rate, and then associates the changes in respiratory rate to the driver’s state of fatigue or drowsiness. Although the idea is very innovative and potentially lifesaving, the system presents a major difficulty: the driver’s arm may cause interference when measuring the respiration based on the chest or abdomen, making the measurement process inaccurate and unreliable.

Mateu-Mateus developed a non-contact system based on the Intel RealSense ZR300 ASV sensor [49]. The small size of the ZR300 module provides a high flexibility for its deployment into a vehicle, which makes the system suitable for this application. The sensor simultaneously uses infrared and depth streams to calculate the driver’s respiratory signal. The driver’s face is initially tracked by applying the optical flow algorithm on infrared frames. Then, the most suitable ROI is detected to extract the respiratory signal based on the depth frames. The algorithm performs in real-time and the system can be used for drowsiness detection while driving in adult population. The experiments’ results showed a good correlation between the acquired respiratory cycles from the proposed system and those recorded by a commercial thorax plethysmography system, used as a gold standard method.

#### 4.1.5. Intensive Care Unit

The intensive care unit is a specific zone in the hospital offering intensive care services for critically ill patients. Admitted patients in intensive care units (ICUs) are closely monitored on a continuous basis. There are two categories of patients in intensive care rooms: ventilated and spontaneous breathing patients. Ventilated patients are usually those facing critical, life-threatening situations, such as respiratory failure. Spontaneous breathing patients present a better condition, but may need to stay in the ICU to promptly provide the necessary care in case of sudden worsening.

While respiration elements are continuously monitored and controlled using mechanical ventilators, there is currently no reliable method to quantitatively assess respiratory function in spontaneous patients. Estimating the tidal volume, for instance, is solely done visually, by the clinician, approximately and subjectively.

Non-contact systems based on cameras can be a very good solution to monitor respiratory volume and detect any breathing disorder in spontaneous breathing patient in ICU. Moreover, non-contact systems should not disturb caregivers’ work or cause distractions that could hurt patients.

A three-dimensional camera-based system has been proposed in [91], for quantitative assessment of spontaneous breathing in the pediatric intensive care environment. The system registers the motion information for the top of the thoracoabdominal surface and the chest lateral regions, performs a surface reconstruction, and then estimates the volume change. However, the respiratory system exhibits a performance difference between small and large volumes, due to the hardware deployed. Indeed, the Kinect’s depth accuracy limit is reached when estimating small volumes between 10 mL and 50 mL.

Nazir et al. [110] recently presented a contactless real time system, based on a single ToF sensor, for quantitative assessment of respiration in ICU patients. By analyzing the patient’s chest wall surface geomorphologic changes, the authors calculate multiple respiratory function parameters: RR and volume. The patient’s torso is automatically detected using a deep neural network model trained on the Common Objects in Context (COCO) dataset, a large-scale object detection, segmentation, and captioning dataset. The validation of the proposed system was performed using both simulated and real patient’s data (16 mechanically ventilated patients admitted in the ICU of the Brest University Hospital). The results were compared to the reference values provided by a mechanical ventilator, which yielded accurate real-time quantitative measures of the pulmonary function.

#### 4.1.6. Other Environments

Mobile devices, such as smart phones, tablets, smart glasses, and smartwatches are increasingly used in respiration research nowadays to capture study endpoints. Indeed, these devices may offer a high-quality medical assistance with their improved cameras’ resolutions, great hardiness, and bearing capacity, being at the same time available, comfortable, and very easy to use. Some papers have proposed to quantify respiration as mentioned in the previous section. Reyes et al. [100] developed a method based on a smartphone to quantify respiratory rate and tidal volume (Table 4). Phokela et al. used an android smartphone and headset microphone to quantify the respiratory rate (Table 1). These two works provided satisfactory results when validated with the ground-truth results, making these systems an advantageous alternative for an assessment that goes beyond the exclusive clinical use. They can be used in a variety of environments such as transport and home environments (Table 4).

Studies in the other environments are interesting to report. For instance, Schumm et al. developed a respiratory system for airplanes [60], which they named the smart airplane seat. Their system directly integrates the respiratory sensor into an airplane seat’s safety belt. The respiration can then be directly correlated with the expansion of the seatbelt. While this work opens many promising avenues towards deploying respiratory assessment systems in airplanes, the technique presents many shortcomings. Passengers must have their seatbelt fastened to meet the system operating conditions. While passengers always have their seatbelts fastened during takeoff and landing, that is not always the case while an aircraft is at cruising altitude.

Another potential application would be the real-time detection of suicide attempts in individual prison cells. Suicide attempts by hanging has been one of the most common suicide methods in correctional facilities in Canada [215]. In [216], authors used a ToF Kinect v2 sensor to represent human movements and model the action of putting the knot from overhead down to the neck. While authors reported that their algorithm achieved 90% accuracy, their system failed to detect some suicidal attempts. One of the main reasons is that some suicidal actions are very similar to some normal activities, such as wearing clothes action, which usually requires moving the hands from bottom to top around the neck. We believe that detecting the respiratory rate using a hidden camera system in prisoner’s individual cell would help to improve their system accuracy and reduce the number of false alarms. In the past, Barland et al. proposed a system to detect deception in prisoners by recording electrodermal, respiration, and cardiovascular activity, and analyze the quantitative evaluations of the physiological responses [217]. The system opens some promising avenues for their deployment in prisons. However, the work presents many disadvantages. First, the subject must be attached to a polygraph. The system also employs a mock-crime paradigm with a prisoner population. Finally, the system require contact with the prisoner, and no quantitative assessment of respiration was reported by the authors. We believe that non-contact systems will be advantageous in correctional facilities.

To summarize the lessons learnt from prior research, each respiration assessment system may operate under certain conditions related to a specific environment. Table 4 shows the common lists of assumptions under which the optimum performance reported in different studies has been achieved. For instance, the low price of electronic components facilitates the deployment of some recent respiratory systems in the user’s home and in sporting environments, as well. By improving the user experience (being contactless and easy to use), researchers (and eventually manufacturers) will allow the use of respiratory assessment in a larger population, including those associated with a lack of cooperation such as children and acute care patients.

Future research should tackle the challenges of developing these systems towards their use in broader and more suitable home applications, making respiration assessment easy, reliable, and not requiring in terms of skilled nursing staff. In clinical settings, accuracy is a crucial constraint, which has important implications on the reliability of patient’s health assessment. In addition to accuracy, real time and continuous monitoring are key parameters in high-risk environments, such as in intensive care units or prison individual cells. Indeed, a short response time is critical for life-threatening events needing immediate intervention. Respiratory systems should be able to evaluate various parameters depending on the environment (home, intensive care unit) and the population (children or adult). Eventually, these systems could include additional features such as automatic interpretation to allow the early response to the caregivers. Low complexity is essential in clinical and intensive care environments. System integration is an important parameter in almost all environments. More compact systems are needed in restricted space settings, such as in vehicles, intensive care environments, etc. The aim is to avoid any disturbance that may be caused by the system itself.

### 4.2. What Are the Limitations on What Can Be Achieved in Respiration Assessment, Using Non-Contact Systems?

Many other parameters are also important for the effective deployment of the respiratory systems proposed in recent studies. These parameters are discussed here, with both an overview of their importance in the different environments and an examination of the tolerance level of each environment. The main parameters include hardware limits, cost, accuracy, and precision, the effects of certain external events, and the patient’s age, sex, and position. Some of these are specific to one environment and are not of crucial importance in other environments.

#### 4.2.1. Spatial Coverage

In imaging- and 3D reconstruction- based works, the number of cameras significantly influences the accuracy of the algorithm. Indeed, the higher the number of camera numbers, the better the spatial coverage of the respiration zone. Until quite recently, researchers had to use at least two cameras to calculate a 3D scene [136,147,218,219]. With the emergence of the new depth cameras [36,37,38,39,40,41], it has become possible to perform 3D reconstruction with only one camera in order to estimate respiratory parameters [6,116,124]. However, researchers still use many cameras in order to improve the spatial coverage [32,91,148] of the respiratory zone, and thereby augment the algorithm’s accuracy and precision.

Accuracy is very important in a clinical environment, where the accuracy and precision of measurements are crucial for patient health assessment. However, a higher number of cameras may be unsuitable for environments, such as intensive care, where the system cannot occupy any additional space, so as not to disturb the patient and health professionals. While the use of a high number of cameras, offers better spatial coverage of the respiratory zone [151,220], covering the whole respiratory zone is not essential when a patient is in their bed.

The zones that reveal respiration motion are the thorax, abdomen, lateral sides, and the back. Covering all of these zones will give a very accurate estimation of respiration. However, covering some parts still gives a good accurate estimation, but with certain limitations, including being sure that the patient does not have a retraction. The abdomen and thorax zones provide more spatial information on respiration than the lateral sides and back. For example, estimating the volume using only the back information will not be enough to estimate tidal volume. To date, using only one camera has not really been enough to accurately cover the whole respiratory zone.

Even if the respiratory information of the back can be neglected, the respiratory information of the lateral sides is essential for a faithful 3D model. Rehouma et al. demonstrated that a minimum of two cameras is enough to cover the breathing region by involving the information of the thoracoabdominal region and the chest lateral parts [32,91]. They compared their method to the reference technique used in pediatric intensive care units. In their findings, they demonstrated that their dual camera-based imaging system provides very high accuracy and precision in estimating the respiratory rate and tidal volume. Indeed, the error between the two methods is very small. However, for greater certainty, it would be worthwhile to conduct the same experiments using four cameras at the bed corners to determine the approximate error between using two and four cameras. Minimizing the camera number adds flexibility to systems-based imaging while accommodating the clinical environments, especially in intensive care units where space occupation has always been a very strict constraint.

#### 4.2.2. Hardware Limitation

Researchers have used the Kinect sensor as data acquisition hardware for many respiratory studies over the years. However, the Kinect sensor was originally developed for a different purpose. Indeed, the first version of the Kinect sensor came with the Xbox 360 for gaming purposes. One hypothesis states that these sensors can be used in medical applications [38]. Another hypothesis states that the Kinect can detect respiratory motion whose magnitude is as small as a few millimeters. The objective was to compare the maximal magnitude detected by the Kinect with the amplitude of respiratory motion and to experimentally validate to what extent this technology can substitute current contact-based solutions [38]. To date, two versions of the Kinect sensor have been used for respiration assessment: Kinect 1 and Kinect 2. Kinect 1 came with the Xbox 360, while Kinect 2 came with the Xbox1 [40]. A description of the depth measurement principle of the Kinect 1, the mathematical model, the calibration parameters, the error sources, and the theoretical error model are presented in [221]. The studies in [222] and [36] offer in-depth comparisons between the two sensors. These works highlight the technological differences between, and the range of data characteristics delivered by the two devices. The stability of the imaging sensors used by the two models (RGB and IR) was compared. While Kinect 2 shows superior performances than Kinect 1, both sensors present depth errors and their noise can be described as second-order polynomial functions. The cameras’ error has been plotted against the distance between the sensor and the scene. The Kinect 2.0 camera is defined by a precision level that decreases linearly with the distance [222]. Based on several experiments, a distance ranging between 1 m and 1.5 m between the camera and the patient has been determined as optimal in the majority of studies. In all cases, a distance greater than 3 m may lead to poor results [197]. Research aiming at understanding the limitations of Kinect sensors have been very helpful in improving algorithm accuracy in respiratory parameter estimation. In [91], the authors have better results for larger volumes than for smaller volumes due to the hardware limitation. Indeed, the Kinect depth camera is not able to accurately detect very small variations of less than 2 mm.

#### 4.2.3. Cost

From a cost perspective, some solutions are more expensive than others, and simply may not be deployable in some environments due to their high cost. On the other hand, the accuracy tolerance level varies from one environment to another. In clinical environments, the cost is generally not very important, but a high accuracy is crucial to avoid skewing of results, especially when a patient is in severe and potentially life-threatening condition. Reliable, innovative solutions providing sufficient accuracy for clinical practice are encouraged regardless of the high costs to deploy such systems. Unlike clinical environments, the cost is very important in-home applications. Therefore, medium accuracy could be accepted if the application is low-cost, as the device could be made more available.

Finding the optimal compromise between accuracy and price would allow such systems to more deployable in such environments. Current depth cameras and mobile phones have the potential for such applications, presenting many advantages such as being portable, low-cost, and contactless. A solution using mobile phones in public transport would be ideal. Making use of an RGB-D sensor, whose cost ranges from $100 to $300, may be a solution for home use, achieving a particularly fine balance between low-cost and accuracy.

#### 4.2.4. Occlusion in Imaging Systems

For several applications, such as continuous monitoring, apnea detection and respiratory-gated whole-body PET/CT imaging, the case of occlusion by a health professional has never been treated in the literature. However, while current imaging systems are not flexible enough to manage such situations, health professionals would only need a few minutes to calculate respiratory parameters. Therefore, such occlusion is not really a disturbing parameter. Nevertheless, we would recommend that the system automatically notify users in case of occlusion. In cases of strict monitoring applications or during surgery where doctors can often cause occlusion, it would be advisable to provide multiple angles of view by using many cameras.

#### 4.2.5. Patient Position Change

During monitoring, the subject may move from his initial position. Wand et al. introduced a novel motion to recognize breathing patterns and detect any sleep apnea using IR data. This method avoids adding restrictions on the patient positioning, allowing patients to sleep on their back or side, with or without facing the camera, completely or incompletely occluded by the bed covers [68]. Using a robust clustering method to identify breathing patterns and irregular respiration, the method achieves high accuracy in detecting apnea episodes and is robust to many occlusion degrees, body poses/movements (i.e., minor head movement, limb movement, body rotation, and slight torso movement), and respiratory behavior (e.g., shallow versus heavy respiration, mouth respiration, thoracic, and abdominal respiration). Nevertheless, patient position change has not been seriously addressed in the literature for the other respiratory parameters, such as tidal volume estimation. In [6] and [92], the subject was in front of the camera in a sitting position during the experimental validation. In [151], the cameras were placed around the patient, who had to remain in a standing position. Unfortunately, the algorithm could not operate if the patient was not in the fixed marked position in front of the camera. A number of studies have designed restraint systems intended to accommodate the patient in a supine [64,130,132,223,224] and prone [142,223] position. Their aim was to accommodate the patient, unlike previous works [151] where the patient stood in a fixed marked position to accommodate the system.

Most works did not treat the position change problem. Some algorithms cannot operate if the patient changes his position, and the others would lose accuracy. For example, accuracy decreases when the patient changes from a supine to a prone position in 3D reconstruction- and imaging- based methods. While observation of the chest and abdomen would provide more surrogate data on respiration, the observation of the lateral sides without chest and abdomen data would provide less accuracy in respiration monitoring, especially when calculating volume. We recommend an investigation to reveal the error when changing position during sleep. In strict real-time monitoring environments requiring continuous monitoring over long periods of time, many of the proposed systems would not be sufficient to give an accurate assessment. In some environments, the applications need at least a few minutes to calculate the respiratory parameters.

#### 4.2.6. Obscuration by Bed Clothing or Bed Sheets

It has been demonstrated that respiratory assessment is easy to detect with a naked torso subject or with a subject wearing lightweight clothes and other types of clothing [75,102,151]. However, the breathing motion is barely perceptible in the case of obscuration by bed clothing or bed covers. Wang et al.’s technique is robust to occlusion by a standard hospital bed cover or sheet [68]. However, the aim of their work was limited to abnormal breathing pattern detection and to recognizing apnea episodes. Moreover, they used infrared data, which are more stable than depth data, making the noise filtering and the detection of subtle breathing patterns easier. Wang et al. did not report any quantitative measures of the physiological parameters, such as respiratory rate, and tidal volume. Some techniques, such as those including 3D reconstruction algorithms, are complex and are not robust enough to function if there is occlusion by a hospital bed clothing or bed cover. Moreover, the lack of distinctive patterns on the bed cover makes use of depth data inefficient. We recommend that more efforts should be made in managing this challenging situation using depth data. For example, the system could notify the user that the results are being distorted by disruptive elements, such as bed sheets.

#### 4.2.7. Real-Time Constraint

As described by Martin et al., a real-time system is a system interacting with an environment by acquiring information, processing/computing them, and giving the results sufficiently promptly to influence the environment at that time [225]. Some respiratory applications reported in the literature are real-time systems, and are very strict in their response time. Other works are not subject to a real-time constraint. Examples of real-time applications include systems used in surgical interventions, apnea detection, sudden infant death syndrome monitoring, and suicide prevention in prisons. In PET acquisition, for instance, the real-time requirement is very strict for artifact avoidance when correcting the respiratory motion [226]. Examples of non-real time applications, also known as batch data processing systems, include unobtrusive approaches for sleep monitoring, in which data collection and processing are two separate steps. These two steps are not necessarily attached or related in time. Once data are collected, they are processed as packages or blocks of data later, which also means the result is received later. Data processing can sometimes take hours or even days. For example, data packages may be processed at night, or the day after data collection, and the subject will get their results only after the processing has been completed.

#### 4.2.8. Number of Respiratory Elements Estimated Simultaneously

In some environments, respiratory applications only estimate one parameter. Other environments require the estimation of many parameters simultaneously. For example, only the respiratory rate is required when driving, and only apnea detection during sleep. The prison environment is another environment where only one respiratory parameter needs to be estimated. Specifically, for suicide prevention applications, the detection of RR may be useful in avoiding false alarms in individual jail cells. In the past, some actions, such as taking off a T-shirt or touching and/or stroking the neck could be confused with the action of setting a rope around the neck [216]. Combining the RR estimation with the ability to detect the setting of a rope around the neck could reduce false alarms or frequent alarm triggering. An example of environments where many respiratory parameters are needed is the intensive care unit, where health professional are observing many parameters together, such as respiratory rate, tidal volume, minute ventilation, and gas exchange (pulse oximetry and transcutaneous carbon dioxide).

#### 4.2.9. Algorithm Complexity

Algorithm complexity is crucial to ensuring a good user experience with a respiratory system. The complexity of an algorithm is highly associated to its processing capacity, accuracy, precision, response time, and computational efficiency. There are two principal complexity metrics of the efficiency of an algorithm: time and space. Time complexity describes the computational complexity, or the amount of time required in relation to the amount of input data, while space complexity describes the amount of memory or space taken by the algorithm in relation to the amount of input data. Several studies are aiming to reduce both the time and space complexity for more efficient and deployable algorithms. Moreover, and as advances continue to be made in technology, the challenging and very complex algorithms of the past are becoming achievable today. Some works were limited to tracking a point in a 2D image, or to using 2D optical flow. Tracking a point/set of points in a 3D scene has become faster and easier. The advent of affordable RGB-D cameras, such as the Kinect sensor, has brought about a profound advancement in scene reconstruction methods [227,228,229], making the whole surface reconstruction and tracking very achievable in real-time, and thus simplifying the volume calculation [91,122].

#### 4.2.10. Age Category

Different age categories have been considered in respiratory assessment works. A number of contactless respiratory systems were designated for adults and did not report results for children and infants. In the case of thorax 3D reconstruction, the particular morphology plays a crucial role in having accurate volume results.

Thorax morphology changes with age and sex. The size, shape, motion magnitude, and velocity are not the same for men, women, and children. For example, neonates tend to have an irregular respiratory pattern that changes between fast and slow, with occasional pauses. Their volumes and flows are much lower compared to adults. For instance, the volumes considered in [100] ranged from 300 mL to 3 L, which does not match with the pediatric volumes. Small children/infants can have a tidal volume as low as 10 mL, but this can reach 500 mL in teenagers. The ventilation requirements in pediatric patients differ from those in adult patients. This has significant potential implications for patient care [230,231,232]. In [91], the accuracy and precision were demonstrated to be lower in smaller volumes compared to larger volumes due to sensor hardware limitations (the depth accuracy limit reached).

#### 4.2.11. Chest Wall Anomalies

The volume calculation methods based on 3D reconstruction and imaging may not give good results in the case of chest wall anomalies, such as retractions and thoracoabdominal asynchrony. Normal thoracoabdominal motion consists of expansion and retraction of the chest and abdomen in the same direction during inspiration and expiration, respectively. Asynchronous thoracoabdominal movement (TAA) is the opposite movement of the rib cage and abdomen during air inhalation [233,234,235]. During TAA, the calculated volume from the 3D reconstruction of the thorax may not correspond to the real inspired/expired volume due to the large pressure difference between the thorax and the abdomen. Indeed, the thorax shape is does not reflect the real inspired/expired volume. While new approaches have been suggested for chest wall spatial motion quantification in critical ill children [160], there is still a lack of methods and techniques to better assess the physiological parameters in patients with acute respiratory failure.

### 4.3. How Can Non-Contact Systems Help Address Some of the Current and Urgent Health Issues in the Present Year (2020)?

Since late December 2019, an outbreak of a novel highly contagious viral coronavirus disease 2019 (COVID-19) pneumonia was reported in Wuhan, China. This virus, which can be deadly to vulnerable people, has spread to almost every country (more than 26 countries worldwide), in a very short time.

Healthcare workers, including physicians, nurses, paramedics, and all other healthcare staff, are facing huge challenges in managing the increasing number of patients. When screening patients, healthcare providers are at high risk of becoming infected and passing on the infection to others. Since the incubation period of coronavirus disease 2019 is around 14 days after exposure, the sick person can be highly contagious without developing clear symptoms of illness. Moreover, the range of COVID-19 symptoms are very similar to those of seasonal flu. One of the most important challenges is how to avoid cross-infection by transmitting the COVID-19 to the care provider, in addition to immediately managing their respiratory failure and other pathophysiological disturbances. Personal protections include two aspects: wearing protective equipment [236] and applying universal precautions, such as keeping at least one-meter distance from a potential sick person [237].

Respiratory systems are very important for identifying patients infected with Covid-19. However, contact methods may expose caregivers to infection while measuring peoples’ vital signs [236,238]. Moreover, all installations, sensors, clothes, and other equipment must be disinfected and/or sterilized after each patient to prevent transmission of disease by either airborne or droplet routes [236]. In a recent study carried out to evaluate the viability of the COVID-19 virus in different environments, it has been shown that the virus is stable on plastic and stainless steel and remains viable up to 72 h after application to these surfaces [238]. Consequently, for these reasons, it is pressing to deploy non-contact systems in clinical environments to minimize risks of the virus transmission. An important requirement to help caregivers in managing COVID-19 patients is to provide them with remote sensing from over 2 m away.

For instance, a new system using radar- and electro-optical-based sensors was announced by Yaakov et al. [96] at the end of March 2020 in Jane’s Defence magazine (Jane’s Information Group Ltd., London, UK), an information company specializing in military, national security, aerospace, and transportation topics. The aim of this project is to identify the persons infected with COVID-19, by remotely measuring their vital signs (pulse, respiratory rate and temperature). The reported system consists of two main modules: a high-resolution perimeter surveillance radar ELM-2114 (ELTA Multi-beam) transmitting and receiving a frequency modulated continuous wave (FMCW) signal combined with an electro-optical sensor. The system designers used the very low frequency K-band to avoid harmful radiation effects on workers and patients. Parameters calculated by the contactless system are the body temperature and inhalation and exhalation rates. The system has a working distance for up to 3 m and can estimate these parameters accurately in real time. The data are sent remotely to the healthcare workers, located in another room.

The system, in its current state, is just an initial step and a first release, designed to respond to an urgent need for protecting healthcare workers. The aim of the radar-based system is mainly to provide a warning/alert to the caregivers of potential infected people. The system provides support and supplements by working alongside existing medical devices rather than replacing them. The next step will involve data processing using advanced computer vision algorithms to remotely provide accurate quantitative measures. Nevertheless, the system in its current release can be deployed in hospitals to answer the urgent need of protecting healthcare professionals. Two trials were conducted and produced accurate results. In the very short term, the company will mainly focus on performing experiments on a big number of patients to move to serial production of the current release. The radar-based system was announced on 31 March 2020 and then was deployed in hospitals, starting from mid-April 2020, for more clinical trials.

In a recent study, Saegusa et al. [48] developed a robot system to detect humans and measure their respiration. The proposed robot incorporates two imaging systems: an Orbbec Astra mobile 3D depth sensor for breathing measurement and a FLIR C2 thermal sensor for human detection. The system is aiming to support the care providers by measuring human volume variation. Different configurations of body postures have been tested. The experimental results showed that the proposed system is accurate to more than 90%. Authors plan to improve the robot interactivity and decision aid ability by adding more advanced features such as breathing anomalies detection.

Many contactless optical systems, based on RGB-D cameras have been previously described by our group in [32,46,91,160]. The experiments were performed for simulated breathing scenarios and for a mechanically ventilated 4-month and 20-day old female, weighing 6.6 kg [91]. Data were collected simultaneously from the proposed system and a mechanical ventilator. For simulated scenarios, different air volumes corresponding to different age categories and weights (ranging from 10 mL to 500 mL) were delivered to the artificial lung by the ventilator to simulate respiration. The performed experiments yielded high accuracy and showed significant agreement with the ventilator, the gold standard method. However, results on the mannequin were slightly better. Relative error between the system RR estimations mean value and RR ventilator value were 3.2516% in the real patient and between 0.99%, and 2.19% for the simulated data.

A contactless system is being developed by our group to identify COVID-19 potential cases, based on the quantitative measures of their respiration. The workload in Sainte-Justine hospital is being intensified, as are the risks to healthcare workers of being infected. The system involves two cameras, a thermal FLIR lepton 3.5 sensor [239] and a 3D Kinect Azure sensor [240]. The two cameras are measuring vital signs in a parallel and complementary way. Using the FLIR sensor, the system can calculate the respiratory rate and the body temperature. In a recent work [241], Lin et al. used a FLIR Lepton 2.5 sensor to continuously measure the human body temperature from a distance up to 0.8 m between the camera and the subject. We aim to improve the system working distance using the next thermal FLIR lepton 3.5 sensor. The respiratory rate is measured as well by tracking the subject’s chest movements using the 3D RGD-D Kinect Azure camera [240]. The heart rate can be calculated by tracking the patient’s face RGB color changes using the Kinect Azure depth sensor. Correlation between the thermic and depth camera is calculated in real time allowing to endorse the accuracy of respiratory rate estimation and to avoid frequent false positives. This real time validation will lead to low false alarm rate, allowing caregivers to positively identify those suffering from shortness of breath while examining all potential COVID-19 infected patients.

The system suggests the latest technologies, such as depth sensing. The sensor is equipped with a 12-MP RGB video camera, providing a very high-resolution color image, with a high frame rate, which can achieve 90 images/second. The sensor includes, as well, a 1-MP depth sensor with wide and narrow field-of-view (FOV) options that help users optimize their applications. Three simultaneous streams can be recorded using an RGB-D Kinect Azure sensor: color, depth, and infrared radiation. The FLIR Lepton 3.5 produces infrared images with embedded temperature readings. Lepton is a complete long-wave infrared (LWIR) camera module conceived to interface easily into native mobile-device interfaces and other electronic devices. Our group is currently working on providing advanced computer vision algorithms allowing real time processing of the recorded data. The healthcare workers will be able to have quantified measures of the respiration and body temperature remotely in the system’s control room, and thus they avoid direct contact with the potential infected patient.

## 5. Conclusions

In this review, we summarized the non-contact-based systems aiming at quantifying the respiration. Non-contact systems have been of increasing interest to researchers and clinicians during the last ten years. A variety of methods have been proposed to provide a practical support or alternative to contact systems, which are not possible to use in all environments and situations, including those associated with a lack of cooperation, such as in newborns, children, elderly people, and acute care patients.

Non-contact technologies include radar detection, cross-sectional sensing, ultrasonic technology, radiography, fluoroscopy imaging, RGB conventional imaging, thermal imaging, and depth sensing, which involves the structured light, time-of-flight, and active stereoscopic vision technologies. Non-contact sensors are aimed at reducing assessment technical complexity, improving a system’s portability, and increasing flexibility. In practice, therefore, the non-contact sensors have reduced the number of expensive components, simplified respiration assessment, and opened the gates to use such systems, not only in hospitals wards, but also in a variety of environments, such as at home, in occupational therapy, and in recreational and sporting environments (e.g., gyms).

During the last five years, camera-based technologies took the greater portion of scientific works aimed to quantify respiration using non-contact techniques. The emergence of low-cost commercial depth cameras has offered next-generation solutions for many complex problems in several fields. Based on the most recent needs in the medical field, our conclusive recommendations for future directions for respiratory system design can be summarized in the following points:

### 5.1. Artificial Intelligence Application

This line of research consists of using the growing knowledge in artificial intelligence techniques in the application of human respiration assessment. Artificial intelligence is intended to improve the scope of the clinical decision-support tools and not to entirely execute tasks that have been traditionally performed by physicians. The key benefits of artificial intelligence over human assessment are its objectivity, scalability, longevity, and continuous improvement capabilities. Moreover, a machine is not susceptible to distractions, sleep deprivation, fatigue, information overload, and short-term memory loss. Such attributes are anticipated to dramatically increase productivity, lower costs, and reduce human subjectivity and error.

Machines are best at digesting massive amounts of data and picking out patterns or seeing things that human brains cannot. Artificial intelligence is reinventing and reinvigorating the organization of the healthcare field through using modern systems that can predict, comprehend, learn, and act.

In an era where both the computation speed and deep neural networks are expanding in a tremendously fast pace, high dimensional unveiled patterns are expected to be leveraged in the extraction of human respiration [242], detecting diseases [243,244], classify apnea events [245], and score illness gravity [160,246]. This direction has recently started gaining attention by researchers [242,243,244,245,246]. However, this innovation continues to be challenged by inherent factors in the healthcare market, making the road to full artificial intelligence integration difficult. Regulators are a key hurdle facing artificial intelligence and machine learning integration, while regulators juggle the balancing act between the advantages and disadvantages of the technology. Data privacy regulation will probably be at the forefront of this battle, leading to a lack of availability of acquisition opportunities in the medical field. Hence, there is not enough validation in many studies. More research databases are needed to train machine learning models.

Another challenge is the lack of medical data documentation. Most medical information, even in electronic health records, is not documented in consistent and discrete ways that can be easily extracted by a computer.

### 5.2. Promoting More Imaging Technology for Data Acquisition

Cameras are revolutionizing the healthcare world, particularly when combined with artificial intelligence algorithms. Measuring respiratory signals from the human body without mechanical contact of the skin has been made possible today using advanced computer vision techniques. This topic has grown rapidly in the last decade. Research approaches were moving towards investigation of many types of imaging data such as color [10,113], depth [91,111,112], and infrared [107,108,114,115,159,247] images. This trend resulted from the emergence of new affordable acquisition devices, such as, Kinect depth sensor [32,46], Orbbec Astra mobile 3D cameras [48,240], RealSense technology [105], and FLIR thermic cameras [48,239]. Such devices are unlocking the next level computer vision applications. In combination with their high accuracy and sufficiently high spatiotemporal resolutions [240,248], 3D cameras have greatly simplified the task of human breath detection, which have given rise to new possibilities for respiratory parameters estimation [6,48,91,122,160].

The goal of a complete and automated diagnosis of respiration can be achieved through this three step-process: first, medical data are acquired using the emerging new cameras. Second, respiratory parameters are extracted from data using image processing techniques. The final step is to endorse the respiratory system capacity to provide an automatic diagnosis of the patient respiration using new machine learning techniques of data analysis such as deep machine learning. However, these new systems are greedy because they require huge sets of training imaging data. More imaging databases are needed to train machine learning models. Future work should be done in this line of research to leverage advancements in imaging data availability in serving the goal of respiratory studies and provide a wider diagnosis of respiratory problems. We encourage creating more synthetic and real patient imaging databases, which can be designed to train machine learning models on how to estimate respiratory rates, volume, blood gases, or breathing disorders and abnormal thoracic movements.

The availability of databases with real patient data has been increasingly hampered by technical barriers related to security and data privacy aspects. Even if some of these barriers were overcome, it is very important to develop and document more synthetic databases. Simulated data are possible and easy to acquire, and results based on them are important to report [46,160].

We encourage to document synthetic databases and find a uniform path to collect medical data in a standardized, quantifiable way that machines can immediately exploit. This lack of imaging databases and their documentation can be addressed through stronger collaboration between engineers and clinicians.

### 5.3. Multidisciplinary Approaches Promoting

Exploring potential capabilities of new technologies in the medical field continue to occupy a growing role in the interventional care of patients with respiratory diseases. This can be mainly achieved through the collaboration between researchers/engineers who design methods and devices, and clinicians who use those devices. This collaboration abides crucial, combining medicine with engineering to provide more effective options for front-line healthcare providers and help them in facing challenges and modern threats to health and society with confidence. Working in multidisciplinary teams will substantially reduce the gap between biomedical research and unmet clinical needs and make promising advances and opportunities in the medical field.

Along with the demand for simpler clinical procedures facilitating the interaction with the patients, the increased concern for sophisticated non-contact medical equipment will further boost demand for biomedical engineers and collaboration between multidisciplinary teams. With growing interest in this field, many systems and methods have been proposed the last few years through merging technological and clinical backgrounds. Many engineers are working on the application of new algorithms and methods to facilitate the diagnosis of illness and pathologies.

However, to be effective, there must be communication between team members through organizing regular meetings, including trainings, the exchange of experience, and interchanging knowledge and points of view for designing a specific medical device. Engineers will always need clinicians to learn about of targeted clinical needs and clinicians need engineers to make optimal use of the technology capability.

Nowadays, many multidisciplinary teams are working together to design new respiration assessment systems, merging both engineering and clinical backgrounds. Encouraging the development of such teams will help in solving the modern medical problems, and thus will improve the clinical care field.

## Figures and Tables

**Table 1:** Overview of some recent research in respiratory assessment over the four last years (from 2020 to 2017).

Year	Author Name, Reference	Respiratory Element	Method/Device (D), Validation Method (V), Validation Dataset or Subjects (S)	Results with Respect to Each Study’s Objective
2020	Lee et al. [44]	Respiratory rate	D: radar sensor. V: respiration belt with peak counting. S: 16 adults	ρ ^1^ = 0.99 (without movements) ρ ^1^ = 0.92 (with weak movements) ρ ^1^ = 0.84 (with severe movements)
	Phokela et al. [45]	Respiratory rate	D: smartphone and headset microphone. V: manual record of inhale and exhale points by users when breathing using an Android application on their smartphone. Users press on a button after each inhalation and exhalation. S: 25 healthy subjects ranging from 10 to 50 years old (11 males and 14 females).	Error = 1% to 9.4% using one source of noise such as television or air conditioner. Error = 1% to 8% in noisy environments (with the combination of various noise sources).
	Rehouma et al. [46]	Respiratory rate Tidal volume Minute ventilation	D: dual-Kinect V2 (3D reconstruction). V: mechanical ventilator. S: a mannequin and 2 infants (1 Male and 1 female).	r^2 2^ = 0.99 in mannequin r^2 2^ > 0.94 in real patients
2019	Reyes et al. [47]	Respiratory rate Respiration movement	D: RGB camera. V: using a reference database comprising 12 video files. S: 4 subjects.	CorrI ^3^ > 90% NRMSE ^4^~10%
	Saegusa et al. [48]	Breathing pattern	D: Orbbec Astra (depth information) + FLIR (Forward Looking InfraRed) sensor C2 (thermal information for person detection). V: Controlled breathing by listening to a metronome set at a frequency of 0.27 Hz over 30 s and by stopping breathing for 20 s for two times. S: 1 subject.	accuracy ~90%
2019	Mateu-Mateus et al. [49]	Respiratory signal	D: Microsoft Kinect V2 (infrared and depth frames). V: Thorax plethysmography system for reference system. S: 20 subjects.	Agreement between the proposed method’s signal and reference method signal: Mean deviation = 10.13 ms No BA ^5^ bias in the BA ^1^ graph p ^6^ < 0.05 global SEN ^7^ = 77.21 % global PPV ^8^ = 80.69 %
2019	Massaroni et al. [10]	Respiratory pattern	D: built-in CCD RGB webcam (iSight camera) integrated into a commercial MacBook Pro laptop (by Apple Inc., Cupertino, California, USA). V: reference respiratory pattern from a head-mounted wearable device recording of the pressure-drop (ΔP) that occurs during the expiratory/inspiratory phases of respiration at the level of nostrils. S: 12 subjects (6 males and 6 females).	Better performance on females: BA ^5^ bias = −0.01 ± 1.02 bpm Females BA ^5^ bias = −0.01 ± 0.73 bpm Males BA ^5^ bias = 0.01 ± 1.22 bpm
2018	Pereira et al. [50]	Respiratory rate	D: infrared thermography. V: long wavelength infrared (LWIR) camera (Vario CAMR HD head 820S/30 mm (InfraTec GmbH, Dresden, Germany)). S: 12 healthy volunteers + 8 newborns.	▪ RMSE ^9^ = 0.31 ± 0.09 BPM (adults) ▪ RMSE ^9^ = 4.15 ± 1.44 (infants)
	Yang et al. [51]	Respiratory rate	D: impulse ultra-wide band (UWB) radar installed in a vehicle. V: a USB pressing button to obtain the ground truth of breathing counts. The button is pressed while a subject is inhaling. S: 4 subjects.	ERROR = 1.06 BPM
	Shoun et al. [52]	Tidal volume	D: thermal data processing. The correlation with the ground-truth measurement is performed using Long-Short-Term-Memory (LSTM) neural network (used as a predictive model for tidal volume estimates). V: spirometry. S: five healthy normal human subjects.	RMSE ^10^ = 10.61%.
2017	Jorge et al. [53]	Breathing pattern (detection of abnormal signals)	D: 3 CCD ^11^ (Sony ICX274AL^®^, Sony, Tokyo, Japan) digital camera (JAI AT-200C^®^, JAI, Glostrup, Denmark). V: a total of 107 events were divided into two independent groups for training and validation and our algorithm was trained to classify true cessations. S: 30 neonatal admissions of less than 37 weeks.	FAR ^12^ reduced of 77.3%
	Liu et al. [54]	Respiration movement	D: wearable strain sensor (WSS). V: measuring tape (MT). S: 21 healthy male students.	ICC ^13^ values for intra-rater reliability were from 0.94 to 0.98 at all locations
	Martinez et al. [55]	Breathing rate	D: depth camera (PS1080, 640x480@30Hz). V: dataset of 3239 segments collected from 67 sleep laboratory patients. S: 67 patients referred to a sleep laboratory with various degrees of sleep apnea.	accuracy = 85.9%

^1^ Correlation coefficient, ^2^ Coefficient of determination, ^3^ The correlation index, ^4^ Normalized root mean squared error, ^5^ Bland-Altman, ^6^ no-correlation coefficient, ^7^ Sensitivity or ratio between TP (True positive) and TP (True positive) + FN (False negative), ^8^ Positive predictive value or ratio between TP (True positive) and TP (True positive) + FP (False positive), ^9^ Root Mean Square error, ^10^ Root-Mean-Square Error, ^11^ Charge Coupled Device, ^12^ False Alarm Rate, ^13^ ICC is a reliability index that shows both degree of correlation and agreement between measurements.

**Table 2:** Examples of works using radar sensors for respiration assessment over the last few years.

Author, Year, Reference	Methods and Results
Kim et al., 2020 [95]	▪ Application of a W-band continuous-wave (CW) Doppler radar sensor ~94 GHz. ▪ Distance from the chest: 1 m. ▪ Application of a low-pass filter. ▪ Frequency of RR signal: 0.27 Hz. ▪ Chest displacement ~0.26 mm. ▪ Good accuracy with the reference manual measurement values.
Islam et al., 2020 [97]	▪ Application of a 24-GHZ phase comparison monopulse radar. ▪ Recognition of multiple human respiratory patterns simultaneously.
Lee et al., 2020 [44]	▪ Technique based on harmonic quefrency selection in the respiration adaptive domain (possible using the nature of respiration signals). ▪ Good correlation with the peak counting validation technique.
Carreiro et al., 2020 [82]	▪ Application of a contactless ultra-wideband (UWB) impulse radar-based sensor. ▪ Application in laboratory and in the Emergency Department settings.
Yaakov et al., 2020 [96]	▪ Application of a Frequency Modulated Continuous Wave (FMCW) radar (ELM-2114) to screen the SARS-CoV-2 virus.
Nosrati et al., 2019 [94]	▪ Application of a phased-array radar. ▪ Use of a hybrid beamforming architecture to generate two simultaneous beams. ▪ Multi-person detection. ▪ The breathing rates of two individuals can be monitored at the same time and using a same frequency (~2.4 GHz).
Yang et al., 2018 [51]	▪ Application of a UWB frequency to detect respiration in a vehicle. ▪ Detection of the minute chest movement. ▪ Mean error ~1.06 breathing rate per minute.

**Table 3:** Comparison of depth sensors.

	Structured-Light (SL)			Time-of-Flight (ToF)		Active Stereo Vision (ASV)		
Parameter/Sensor	Microsoft Kinect v1	ASUS Xtion	Orbbec Astra S	Microsoft Kinect v2	Kinect Azure DK ^3^	Intel R200	Intel D415	Intel D435
Frame Rate (FPS ^1^)	30	30	30	30	5–15–30	90	90	90
Color Resolution (px ^2^)	640 × 480	SXVGA ^4^ (1280 × 1024)	1280 × 960 @ 7 FPS 640 × 480 @ 30 FPS 320 × 240 @ 30 FPS	1920 × 1080	Up to 3840 × 2160	1920 × 1080	1920 × 1080	1920 × 1080
Depth Resolution (px ^2^)	640 × 480	VGA ^5^ (640 × 480) QVGA ^6^ (320 × 240)	VGA ^5^ (640 × 480) QVGA ^6^ (320 × 240) QQVGA ^7^ (160 × 120)	512 × 424	NFOV ^8^ unbinned 640 × 576 NFOV ^8^ 2 × 2 binned (SW ^9^) 320 × 288 WFOV ^10^ 2 × 2 binned 512 × 512 WFOV ^10^ unbinned 1024 ×1024 Passive IR ^11^ 1024 × 1024	640 × 480	1280 × 720	1280 × 720
Field of view	57° × 43°	57° × 43°	60° × 49.5°	70° × 60°	Up to 90° × 59°	59° × 46°	63.4° × 40.4°	85.2° × 58°
Range (meter)	0.8–4.0	0.8–4.0	0.4–2.0	0.5–4.5	0.25–5.46	0.5–6.0	0.16–10	0.2–4.5

^1^ Frame per Second, ^2^ Pixels, ^3^ Developer Kit, ^4^ Super eXtended Video Graphics Array, ^5^ Video Graphics Array, ^6^ Quarter Video Graphics Array, ^7^ Quarter-Quarter Video Graphics Array, ^8^ Narrow Field-of-View, ^9^ Short Wave, ^10^ Wide Field-of-View, ^11^ Infrared.

**Table 4:** Summary of recent respiratory systems and their applications.

Year	Author Name, Reference	Respiratory Element	Method/Device (D), Validation Method (V), Validation Dataset or Subjects (S)	Results with Respect to Each Study’s Objective	Environment/ Applications
2020	Chen et al. [99]	Respiration rate	D: Doppler and passive radio sensing. V: video recordings. S: 1 subject with four testing positions.	▪ Average Phase Variation between 0.8 and 1.05	Home Environments ▪ Quality of sleep assessment. ▪ Apnea detection. ▪ Sudden infant death syndrome (SIDS). ▪ Children and neonates’ health supervising. ▪ Respiration monitoring.
	Schätz et al. [111]	Respiratory pattern	D: depth data processing from a variety of depth sensors (MS Kinect v2, RealSense SR300, R200, D415, and D435). V: a neural network classifier (simple competitive NN) was trained on a set of whole night polysomnographic records with a classification of sleep apneas by a sleep specialist. S: 57 patients (32 healthy and 25 patients having sleep apnea).	▪ Precision ~95.4%	
2019	Delimayanti et al. [157]	Respiratory pattern and breathing activities	D: depth data processing from Kinect v2, using FFT ^1^ & PCA ^2^. Then, a classification is performed using SVM ^3^ classifier. V: cross-validation using complementary subsets (learning + testing). S: 4 subjects with 10-fold cross validation.	▪ Accuracy ~90%	Clinical Environments ▪ Health assessment. ▪ Respiration and vital signs monitoring.
2018	Rehouma et al. [91]	Respiratory rate Tidal volume	D: dual-Kinect sensor (surface reconstruction). V: mechanical ventilator. S: mannequin + 1 patient.	Respiratory rate: ▪ (RE ^4^ = 3.25%, RSD ^5^ = 9.87%) ▪ Tidal volume: ▪ (RE ^4^ = 9.17%, RSD ^5^ = 12.3%)	Intensive Care Unit ▪ Respiration monitoring in spontaneous breathing patients. ▪ Breathing disorder detection.
2017	Aoki et al. [103]	Minute ventilation (VE)	D: extraction of motion waveform using a Kinect v2 sensor under a high exercise intensity of ≥100 W. V: expiration gas analyzer. S: 6 subjects.	▪ ventilation threshold = ±10 W ▪ p ^6^ < 0.001 ▪ ρ ^7^ > 0.79 ▪ Maximum Bias ± 95%, CI: 0.1119 ± 0.33.	Sport ▪ Athletic performance improvement. ▪ Track and measure breathing during training.
	Schoun et al. [52]	Tidal volume	D: measures are obtained from thermal images, correlated with ground-truth measures, and then trained into a recurrent network model using TensorFlow library. V: spirometry. S: 5 subjects.	▪ RMSE ^8^ = 10.61% (tidal volume). ▪ RMSE ^8^ = 21.81% (raw flow signal).	Clinical/Home Environments ▪ Lung conditions monitoring. ▪ Health assessment.
	Sharp et al. [122]	Respiratory function testing	D: 3D reconstruction of the subject’s thorax using depth data of a Kinect v2. V: spirometer, S: 251 recorded efforts.	▪ FVC ^9^ (r = 0.999, p < 0.001) ▪ FEV1 ^10^ (r = 0.937, p < 0.001) ▪ VC ^11^ (r = 0.998, p < 0.001) ▪ IC ^12^ (r = 0.995, p < 0.001)	Clinical Environments ▪ Monitoring of disease severity and progression by performing PFT ^13^.
	Soleimani et al. [6]	Forced vital capacity measures	D: chest wall surface reconstruction using depth data of a ToF ^13^ sensor. V: spirometer. S: 85 patients.	r^2 14^ = 0.98	Clinical Environments ▪ Assessment and detection of respiratory pathologies.
2016	Ripoll et al. [98]	Respiratory rate	D: chest wall surface reconstruction using depth data of a ToF ^13^ sensor. V: data recorded by a plethysmography band. S: 5 subjects.	α ^15^ = 0.99	Vehicle (driving) ▪ Fatigue indicator. ▪ Alcohol and drug-impaired driving detection. ▪ Driver drowsiness detection
2016	Reyes et al. [100]	Respiratory rate Tidal volume	D: estimation of a volumetric surrogate signal on a smartphone. Authors analyze the intensity changes in the video channels caused by the chest wall movements during breathing. V: spirometry. S: 15 subjects.	▪ Respiratory rate (r^2 13^ = 0.99, RMSE ^8^ = 0.414 + 0.178 bpm) ▪ Tidal volume (r^2^ ^13^ = 0.95, RMSE ^8^ = 0.182 ± 0.107 L)	Transport/Home ▪ Detection of respiratory arrest. ▪ General health assessment.
	Reinaux et al. [123]	Tidal volume	D: optoelectronic plethysmography (OEP). V: comparison with pneumotachograph data. S: 20 infants.	▪ Mean Vt ^16^ difference ~0.02 mL, ▪ Limit of agreement 4.11 to 4.08 mL (95% CI ^17^), ▪ Contribution to V ^18^_T, OEP_ ▪ 12.4 ± 9.7% (pulmonary rib cage) ▪ 5.2 ± 5.1% (abdominal rib cage) ▪ 82.4 ± 11.4% (abdomen)	Clinical/Home environments in infants. ▪ Health assessment. ▪ Respiration and vital signs monitoring.
	Procházka et al. [93]	Respiratory rate	D: video sequences of thorax movements are recorded by MS Kinect sensor to enable their time analysis in selected regions of interest. V: contact-based sensor (Garmin Ltd.). S: record of 120 s of image, depth, and infrared video frames.	▪ Accuracy ~0.26%	Home environment ▪ Health assessment. ▪ Quality of sleep assessment ▪ Diagnosis of obstructive sleep apnea severity. ▪ Diagnostic of physical activities.
2016	Sirevaag et al. [101]	Respiratory rate, Respiratory pattern	D: laser Doppler vibrometry (LDV). V: data from Biopac SS5B circumferential belt, at a lower thoracic level. S: 32 healthy participants.	▪ ρ ^7^ = 0.99	Harsh environments (e.g., including the MR scanner where the laser head can be separated from the magnetic field). Clinical environments.
	Ostadabbas et al., [92]	Airway Resistance Tidal volume	D: depth data processing of a segmented ROI, called chest bounding box. The segmentation is performed to optimally demonstrate the lung volume changes during respiration. V: clinical results using spirometry and plethysmography tests. S: 14 patients.	▪ Tidal volume error = 0.07 ± 0.06 L. ▪ Accuracy in predicting three levels of severity of airway obstruction = 76.2%. ▪ Accuracy of airway obstruction detection = 80%.	Clinical/Home environment ▪ Lung conditions monitoring. ▪ Obstructive pulmonary disease detection. ▪ Asthma detection. ▪ Respiration monitoring.
2015	Heß et al. [148]	Abdominal and thoracic patterns	D: 3D reconstruction based on two Structured light cameras data. V: moving a high-precision platform with 10-micrometer accuracy. S: 10 patients.	▪ Abdominal region: ρ ^7^ = 0.74 ± 0.17 ▪ Thoracic region: ρ ^7^ = 0.45 ± 0.23	Clinical environment ▪ Breathing disorder detection. ▪ Respiration monitoring.
2014	Tahavori et al. [141]	Respiratory motion	D: multi-ROI analysis, to investigate the dominate variations using PCA, based on depth data from a structured light sensor. V: multi-ROI analysis performed on 3 separate sessions. S: 20 subjects.	The first principal component describes more than 70% of the motion data variance in thoracic and abdominal surfaces.	Clinical environment ▪ Abnormality detection. ▪ Analysis of disease severity.
2014	Benetazzo et al. [75]	Respiratory rate	D: a system based on structured light sensor detects the human chest and calculates its distance from the camera to predict the respiratory rate. V: spirometry. S: 5 subjects.	▪ p ^6^ < 0.001 ▪ ρ ^7^ > 0.92	Sitting person in an indoor environment such as clinical environment, home environment.
2010	De Boer et al. [119]	PFT ^19^ changes (FEV ^10^, FVC ^9^)	D: structured light plethysmography (SLP) based on structured light cameras. V: spirometer and pneumotachograph data. S: 40 patients.	▪ r^2 14^ = 0.91 (volume). ▪ r^2 14^ = 0.97 (forced expiration).	Anesthesia and intensive care environments.

^1^ Fast Fourier Transform, ^2^ Principal Component Analysis, ^3^ Support Vector Machine, ^4^ Relative Error, ^5^ Relative Standard Deviation, ^6^ no-correlation coefficient, ^7^ correlation coefficient, ^8^ Root mean squared error, ^9^ Forced vital capacity, ^10^ Forced expiratory volume of 1 s, ^11^ Vital capacity, ^12^ Inspiratory capacity, ^13^ Time-of-Flight, ^14^ Coefficient of determination, ^15^ Cronbach’s alpha coefficient, ^16^ Tidal volume, ^17^ Confidence Interval, ^18^ Measurements of tidal volume by Optoelectronic plethysmography, ^19^ Pulmonary Function Test.

**Table 5:** Assumptions under which the optimum performance reported in different literature works are achieved.

	Home Applications	Clinical Environment	Sporting Environment	Vehicles	Intensive Care Environments	Prisons	Universal
Low-cost	✓✓	✗	✓✓	✓	✗	✗	✓
Continuous monitoring	✗	✓	✗	✓	✓✓	✓✓	✗
Non-contact	✓	✓	✓	✓	✓✓	✓✓	✓✓
Integration in environment	✓	✓	✓	✓✓	✓✓	✓✓	✓✓
Real-time	✗	✓	✗	✓✓	✓✓	✓✓	✓✓
High accuracy	✗	✓✓	✗	✓	✓✓	✓✓	✓✓
Many respiration parameters	✗	✓✓	✗	✗	✓✓	✗	✓
Results self-interpretation ^1^	✓✓	✗	✓	✓✓	✗	✓✓	✓
Low complexity	✓✓	✓✓	✓✓	✓✓	✓✓	✓✓	✓✓
Low space occupation	✓	✓	✓	✓✓	✓✓	✓✓	✓
Embedded processing ^2^	✗	✗	✓✓	✓✓	✓	✓✓	✓✓
Mobility	✗	✗	✓	✗	✓✓	✗	✓✓
Demanding high user experience ^3^	✓✓	✓✓	✓✓	✓✓	✓✓	✓✓	✓✓
Network availability (Wi-Fi)	✗	✗	✗	✗	✓✓	✓✓	✓✓

✓✓ indicates that the constraint is absolutely required for deployment in the corresponding environment; ✓ indicates that the constraint is required for the deployment in the corresponding environment; and ✗ indicates the constraint is not required or is optional for the effective deployment in the corresponding environment. ^1^ Results are comprehensible and accessible to users, ^2^ Not connected to a computer. ^3^ Comfortable and easy to use without the guidance of qualified personnel.

**Figure A1:**
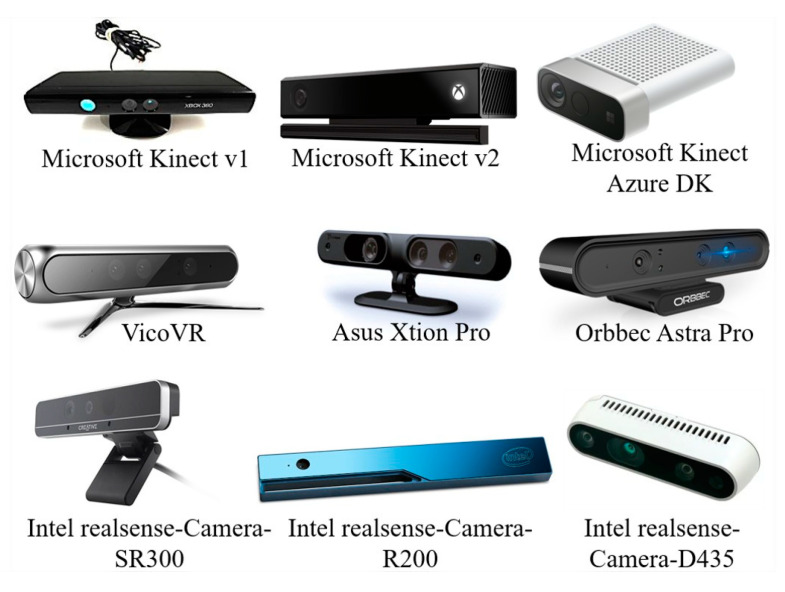
Recent RGB-D sensors: structured-light (Kinect v1, Asus Xtion Pro, Intel SR300), time-of-flight (Kinect v2, Kinect Azure DK, VicoVR), and active stereo vision (Intel R200, D435).

## Appendix A. Summary of Respiratory Elements

### Appendix A.1. Respiratory Rate

Respiratory rate (RR), also called Breathing Rate is one of the principal signs used to assess the vital function of the human organism [249]. Expressed and measured in terms of breaths per minute (BPM), the respiratory rate provides an early indication about a patient’s health state and helps to monitor the progression of illness. The normal range of respiratory rate depends on a subject’s age. On average, RR is between 12 and 20 BPM in normal adults, 15 and 30 BPM in children, and can be as high as 30 to 60 BPM in infants and neonates [250]. Extreme variations in RR, either low (called bradypnea [251,252]) or high (called tachypnea [251,253]), may predict life-threatening events [76]. Even though RR is often used as indicator of various respiratory dysfunctions, it can also be used to assess human stress and emotions [254,255].

### Appendix A.2. Respiratory Volumes

Measuring lung respiratory volumes is a part of a pulmonary function test (PFT) and is helpful in-patient diagnosis, treatment, and monitoring. In hospital wards, the gold standard techniques are based on spirometry and body plethysmography. A spirometer is a machine including a mouthpiece hooked up to a small electronic device. In body plethysmography, the patient is asked to sit or to stand inside an airtight box. These instruments require patient cooperation to estimate clinically relevant lung volumes, forced expiratory, and inspiratory flows. The most common parameters considered in spirometry and pulmonary function tests are listed in Table A1.

**Table A1:** A summary of the common parameters considered in spirometry and PFT ^1^.

Physiological Parameter Description	Acronym
Vital capacity	VC
Tidal volume	Vt
Forced vital capacity	FVC
Functional Residual Capacity	FRC
Forced expiratory volume at timed intervals of 0.5 s after full inspiration	FEV
Forced expiratory volume of 1 s	FEV1
Forced expiratory volume at timed intervals of 2 s	FEV2
Forced expiratory volume at timed intervals of 3 s	FEV3
Forced expiratory flow 25–75%	FEF 25–75
Maximal voluntary ventilation or Maximum breathing capacity	MVV
Expiratory reserve volume	ERV
Inspiratory capacity	IC
Inspiratory vital capacity	IVC
Total lungs capacity	TLC

^1^ Pulmonary Function Test.

Spirometry is the most reliable way to test patients’ lung function in chronic pulmonary disease (COPD, cystic fibrosis, asthma…). The airflow is measured through electronic and mechanical concepts. The system uses electronic devices, namely a microprocessor for data processing and a recorder for registering patient results and displaying them on a graph. Other derived parameters may be calculated in certain situations, such as the Tiffeneau–Pinelli index (FEV1/FVC) in restrictive diseases, defined as the ratio of the forced expiratory volume in 1 s (FEV1) to the full-forced vital capacity (FVC).

Body Plethysmography, also known as body box, is well-established as a reference method in clinical environments and is the preferred approach for calculating a variety of respiratory volumes such as the forced vital capacity and total lungs capacity [256]. The whole-body plethysmograph consists of a rigid, sealed, and restricted chamber, in which subjects may be asked to be in a sitting or standing position while breathing through a pneumotachograph. Pressure transducers of different sensitivity are fixed to estimate the pressure and volume across the pneumotachograph. This technique is only available for stable patients and cannot be used in an intensive care environment or for young children.

### Appendix A.3. Blood Gas Concentrations

Gas exchange assessment is most often done by measuring oxygen partial pressure (PO_2_) and carbon dioxide partial pressure (PCO_2_) in the blood, either arterial, venous, or capillary blood. The level of PCO_2_ gives an adequate indication of alveolar ventilation, decreasing in alveolar ventilation results in an increase of PCO_2_. When measured in the arterial blood, a decrease in the PaO_2_ level is a good indicator of severe acute respiratory disease. The blood pH, also measured in gas blood tests, is maintained within a narrow range interval [7.35–7.45] in healthy patients. The bicarbonate level (HCO3-) in blood is a part of the gas blood test, and, combined with the pH level, it guides the physician to perform his or her diagnosis. Gas blood tests are invasive measures, but other non-invasive tests are available for monitoring, such as transcutaneous oxygen saturation levels, end-tidal PCO2, transcutaneous PO_2_, and PCO_2_. Measurement of transcutaneous oxygen saturation is performed through pulse oximetry. End-tidal PCO_2_ is measured in the expiratory air, and transcutaneous PO_2_ and PCO_2_ are measured using skin sensors and are commonly used in neonatology. Efficient ventilation will result in optimal gas exchange, and so gas exchange assessment will offer a useful clinical information to physicians.

### Appendix A.4. Chest Wall Motion

Chest wall motion assessment is an essential clinical element in monitoring patients with respiratory problems and is a main part of the estimation of the severity of a disease, as illustrated by the number of clinical scores (m-Wood, Pediatric Respiratory Assessment Measure (PRAM), etc.) that include this assessment [257,258,259]. However, it is currently only a clinical assessment (visual inspection) and thus is subject to high subjectivity. Chest wall assessment includes not only thorax and abdomen amplitude estimation, but also the relative movements of the ribcage compartments, deformities quantification, and abnormal pattern identification, such as thoracoabdominal asynchrony and other respiratory muscle retraction [1,233,235,260,261,262,263,264,265].

The chest wall assessment was investigated in many works, to detect the disease severity. Respiratory failure (RF) is a major reason for intensive care unit (ICU) admission in children and adults. RF is characterized by shortness of breath, poor oxygenation, and increased work of breathing (WoB). The chest movement is most often a sign that the patient has a breathing disorder. Intercostal retractions, for example, occur when the muscles between the ribs pull inward due to primary muscles workload. This leads to the activation of the accessory muscles to meet ventilation demands. Necessity of mechanical ventilation is currently decided by qualified clinicians, through visual inspection, to prevent RF from progressing to respiratory arrest. However, human assessment is subjective and is practically impossible to audit.

## Appendix B. Overview on Non-Contact Technologies

### Appendix B.1. Radar Sensors

Radar systems transmit and receive radio waves, whose frequencies depends on the application requirement. In the electromagnetic spectrum, radio wavelengths are longer than infrared light. Radar systems include both emitters and receivers. Emitters transmit the electromagnetic signal, and the receivers decode the received signal. The phase shift *θ* is given by Equation (A1), which is characterized by a wavelength λ and a traveled distance *d*. Equation (A2) illustrates the total received signal, where a is the attenuation coefficient for each path and *i* is the path identifier.

(A1) θ=2πλ/λ

(A2) $$\sum^{\;}a_{i}e^{-j2\pi d_{i}/\lambda }$$

Radar systems have frequencies from 300 GHz to as low as 3 kHz, with corresponding wavelengths from 1 mm to 100 km. The choice of frequency used depends on the radar application requirements. The minimum antenna size is proportional to wavelength and inversely proportional to frequency. Remote radar-based sensors are mostly used to detect physiological motion by demodulating and analyzing the signal reflected off the thoracoabdominal wall. Doppler-based radars were particularly attracting many researchers last years due to their high precision. This category is based on the Doppler effect to produce velocity data using a microwave signal off the thoracoabdominal wall and analyzing how the former’s motion has altered the frequency of the returned signal.

### Appendix B.2. Cross-Sectional, Ultrasound, Radiography, and Fluoroscopy Imaging Sensors

Cross-sectional imaging is usually used to refer to Computerized tomography (CT), Magnetic resonance imaging (MRI), Positron Emission Tomography (PET), Single-Photon Emission Computerized Tomography (SPECT), and related imaging techniques that view the body in cross-section, i.e., as axial (cross-sectional) slices.

Even though ultrasonography generates two-dimensional image “slices” of the body, some clinicians trend to not classify it as a cross-sectional technique. The reason is that the angle of the slices is often not perpendicular to the axis of the body. Ultrasound sensors use sound waves, which are reflected, deflected, or absorbed in the human body. The ultrasound image is constructed using the reflected sound waves. The ultrasound images have been a good alternative to cross-sectional imaging techniques for tracking respiration and organs moving with respiration [144,266].

A few works have used 2D, 3D, and 4D ultrasound images, which are all based on sound waves to create the image. The old ultrasound standard uses 2D imaging, which is a series of flat, 2D cross-section gray scale images of the scanned tissue. 3D ultrasound allows the scanning of tissue cross sections at different angles to get a three-dimensional representation through data reconstructing techniques. The 4D ultrasound uses time as the fourth dimension to add movement and offer a more realistic representation.

Examples of imaging techniques that are not cross-sectional include plain radiography, and fluoroscopy. The techniques rely on projection of an x-ray beam through an object to a receptor. Planar nuclear medicine is also not cross-sectional, although the imaging radiation is emitted from the object of interest, rather than passed through it.

### Appendix B.3. RGB and Thermal Sensors

The RGB color model considers that any color can be made by combining (R), green (G) and blue (B). RGB sensors refer to devices acquiring real world objects and projecting them on a two-dimensional (2D) view plane (i.e., the sensory surface). One of the most famous models used by a variety of RGB sensors is the Pinhole Camera model, which describes the mathematical relationship between the coordinates of a point in 3D space and its projection onto the 2D image plane.

The RGB sensors usually record compressed RGB video based on computer vision methods. An RGB video is a sequence of successive frames. Each RGB frame, also called intensity frame, is composed of three images (matrices of pixels) in the red, green, and blue channels. The pixel, abbreviated as “px”, is the unit of measurement used in digital images. Each pixel is encoded as a number from 0 (black) to 255 (white) and the size of the image matrix varies with the resolution of the camera used for acquiring data.

The advantage of using RGB sensors-based techniques is that they present lower cost, fast acquisition, and higher resolution over time. Computer vision techniques have improved significantly and are considerably reducing the noise in acquired digital images. Nevertheless, 2D images cannot fall outside the projection model, which means bringing some part of the real-world objects into sharp focus at the expense of blurring other parts and obscuring some details.

Measures of thermal changes have been used to monitor respiration rate with infrared (IR) video [59,65,187,188,189,190,191,192,193]. Cameras based on infrared thermography monitor dynamic thermal activity emitted from specific areas of the body and have been applied to monitor human physiological signals.

### Appendix B.4. Depth Sensors, SL, ToF, and ASV Technologies

An RGB-D (Red, Green, Blue, Depth) sensor, also simply called depth sensor, includes three embedded sensors: an RGB color camera (for color images acquiring), an infrared radiation-emitting laser and an infrared sensing camera (for infrared and depth images sensing). With the introduction of low-cost PrimeSense consumer sensors in 2010, good quality color and depth data can be easily acquired simultaneously using a single sensor, at high frame rates and affordable cost. Traditional depth images were essentially based on passive stereo vision (PSV) techniques, which requires at least two calibrated RGB cameras. Calibration of the internal and external parameters of stereo vision cameras is a classical research problem in the computer vision sphere and is still too complex and lengthy. Using RGB-D sensors, it is made possible to acquire depth data with lower time and costs.

Depth sensors rely on different technologies, such as structured light (SL), time of flight (ToF), active stereo vision (ASV).

Structured-light techniques use a light source with diverse projection patterns to calculate variations in the observed surface. The depth map is provided by analyzing the deformation of the pattern acquired by the sensor (against the projected one, which is known). SL sensors operates well in capturing depth data at high frame rates up to a range of 1–3 m indoors.

Time-of-Flight techniques calculate, first, the total time-of-flight required for a single laser pulse to travel from the sensor to the surface and then to come back to the sensor after being reflected by the surface. The distance between the sensor and the surface is then deducted from this time difference using Equation (A3):

(A3) $$d=\frac{\Delta \varphi }{4\pi f}\times c,$$

where d is the distance to be measured (pixel depth), Δφ is the phase shift between the emitted light and the reflected light, c is the speed of light (3 × 108 m/s) and f is the modulation frequency. The accuracy of ToF sensors is better than SL sensors in outdoor environments.

Active Stereo Vision sensors is combining both stereo vision and structured-light techniques. By embedding an active light source emitting a known pattern, ASV systems offer an alternative approach to the use of two cameras. Thus, the stereo matching problem is simplified and directly solved.

Microsoft Kinect v1, one of the first consumer depth sensors, was based on PrimeSense SL technology. Initially introduced for gaming, The Kinect v1 became a very popular sensor for computer vision. By providing dense 3D information at high frame rates (30 Frames/second), Kinect v1was used for a variety of fields and applications such as tracking and recognition [267,268,269,270]. By 2015, Microsoft announced its second generation of the Kinect family with the introduction of the Xbox One game console: the Kinect v2. Using the Time-Of-Flight (ToF) technology, the Kinect v2 sensor presented improved performances compared to its SL version, especially in outdoor environments. Kinect v2 was used for a variety of applications such as mobile robot navigation [198], human motion recognition [271], and suicide attempts preventing [272].

In turn, Intel manufactured its own family of RGB-D cameras using the SL and the ASV technologies. The Intel F200 and SR300 (2015) are based on SL technology, while the R200 (2015), D415 and D435 (2018) are based on active stereo vision (ASV), which utilizes a near infrared (NIR) texture emitter paired with two NIR sensors.

Besides, a variety of improved consumer depth sensors have been released by several companies over the last years. In Figure A1, examples of affordable consumer RGB-D are given, such as the last Microsoft Kinect Azure (2020), Asus Xtion Pro Live (2011), VicoVR (2017), the Intel RealSense D435 (2018), and the Orbbec Astra Pro (2015).

The main drawback is that of commercial depth sensors work with a borderline level of acceptance of depth resolution. From year-to-year, this level is being improved with the advent of new technologies.
